# Impact of tobacco and/or nicotine products on health and functioning: a scoping review and findings from the preparatory phase of the development of a new self-report measure

**DOI:** 10.1186/s12954-021-00526-z

**Published:** 2021-07-30

**Authors:** Esther F. Afolalu, Erica Spies, Agnes Bacso, Emilie Clerc, Linda Abetz-Webb, Sophie Gallot, Christelle Chrea

**Affiliations:** 1 PMI R&D, Philip Morris Product S.A., Quai Jeanrenaud 5, 2000 Neuchâtel, Switzerland; 2 Patient-Centered Outcomes Assessments Ltd., 1 Springbank, Bollington, Macclesfield, Cheshire, SK10 5LQ UK

**Keywords:** Health and functioning, Scoping review, Tobacco and/or nicotine products, Modified risk tobacco products, Qualitative research, Conceptual framework

## Abstract

**Background:**

Measuring self-reported experience of health and functioning is important for understanding the changes in the health status of individuals switching from cigarettes to less harmful tobacco and/or nicotine products (TNP) or reduced-risk products (RRP) and for supporting tobacco harm reduction strategies.

**Methods:**

This paper presents insights from three research activities from the preparatory phase of the development of a new self-report health and functioning measure. A scoping literature review was conducted to identify the positive and negative impact of TNP use on health and functioning. Focus groups (*n* = 29) on risk perception and individual interviews (*n* = 40) on perceived dependence in people who use TNPs were reanalyzed in the context of health and functioning, and expert opinion was gathered from five key opinion leaders and five technical consultants.

**Results:**

Triangulating the findings of the review of 97 articles, qualitative input from people who use TNPs, and expert feedback helped generate a preliminary conceptual framework including health and functioning and conceptually-related domains impacted by TNP use. Domains related to the future health and functioning measurement model include physical health signs and symptoms, general physical appearance, functioning (physical, sexual, cognitive, emotional, and social), and general health perceptions.

**Conclusions:**

This preliminary conceptual framework can inform future research on development and validation of new measures for assessment of overall health and functioning impact of TNPs from the consumers’ perspective.

**Supplementary Information:**

The online version contains supplementary material available at 10.1186/s12954-021-00526-z.

## Background

As a leading cause of preventable morbidity and mortality worldwide, smoking remains a major public health problem. Compared with those who do not smoke, people who smoke are significantly more likely to develop heart diseases, lung cancer, chronic obstructive pulmonary disease (COPD), and other diseases [1, 2]. It is well established that the best way to avoid the health risks associated with smoking is for people to never start and for those who smoke to quit [1, 3]. Tobacco harm reduction is one way to alleviate the health risk for individuals who choose not to quit smoking [4], by providing less harmful tobacco and/or nicotine products (TNP), such as reduced-risk products (RRP) (used here to refer to products that present, are likely to present, or have the potential to present, less risk of harm to people who smoke and switch to these products versus continued smoking) or modified risk tobacco products (MRTP).

Several smokeless tobacco products and a heated tobacco product were recently authorized for marketing with modified risk claims through the United States (US) Food and Drug Administration (FDA) MRTP pathway [5]. The guidance on MRTP applications [6] specifies that health outcomes should be assessed during premarket evaluation and postmarket surveillance of modified risk TNPs such as these. These health outcomes comprise not only objective clinical and biological measures but also self-reported outcomes [6, 7]. Studies and reports have recently started providing evidence on the health impact of new TNPs [8]. For instance, recent papers have investigated the effects of e-cigarettes and heated tobacco products on cardiopulmonary outcomes [9–14]. However, the papers have mainly focused on clinical measurements, such as spirometry and other lung function tests; consumer perception is rarely explored or the focus of the research. Measuring self-reported experience is important for understanding the changes in the health status of individuals switching from cigarettes to RRPs and is a key component of tobacco harm reduction strategies [7]. Self-reported ratings of RRP effectiveness or adverse events might differ from clinical measures and provide another perspective as useful as the clinicians. In addition, consumer perception of positive changes in health status, functioning and other behavioral outcomes will also subsequently influence use behaviors and switching to RRPs rather than continuing smoking.

Self-perceived health status is a complex concept to define and measure, particularly within the context of TNP use [15]. While generic health status measures, such as the Medical Outcomes Study 36-item Short-Form Health Survey (SF-36), have been used to evaluate the health status of people who smoke [16, 17], comparisons have mainly been made between those who currently smoke, those who used to smoke, and those who never smoked [18, 19]. Results from these studies strongly suggest that, in healthy populations, existing generic measures are not sensitive enough to detect change over time when stopping or switching from cigarettes to other TNPs, owing to high ceiling effects [20]. While a few smoking-specific quality of life measures have been developed, these measures have not been widely implemented or standardized [15, 17, 21, 22], and the application of these smoking-specific measures to different TNP use across the risk continuum is scarce [20].

As part of the **A**ssessment of **B**ehavioral **OU**tcomes related to **T**obacco and Nicotine Products (ABOUT™) Toolbox initiative [23], the present project aims at developing a new self-report measure (ABOUT™—*Health and Functioning*) to address the current gap and assess the impact of TNPs on health and functioning (including health status, functional status and other health-related quality of life constructs). This paper presents insights from three research activities [24, 25] from the preparatory phase of development of the measure—that is, a scoping literature review, reanalysis of consumer focus groups/interviews, and expert opinion. These three activities serve as background research to support the development of a preliminary conceptual framework of health and functioning associated with the use of TNPs.

## Methods

### Scoping literature review

The purpose of the review was to address two main questions among individuals who use TNPs:

- What are the positive and negative health and functioning impacts of TNP use?
- What concepts are evaluated by measures used to assess the positive and negative impacts of TNP use?

Given the nature and breadth of the research questions and the number of potentially relevant publications, a scoping literature review was used as it provides a means of identifying the literature and mapping the concepts and evidence on a topic by using an informative and iterative research process [26]. The scoping review involved a PubMed search (August 2018) and application of Sciome’s rapid Evidence Mapping (rEM) [27], followed by additional manual screening and review. rEM is a proprietary methodology developed by Sciome (https://www.sciome.com/) to rapidly summarize and produce a quantitative representation of the available body of scientific evidence in a particular area. The study by Lam et al. demonstrated a proof-of-concept application of the rEM methodology [27]. The PubMed search terms targeted qualitative and quantitative research among people who use TNPs (Table 1). This was supplemented by a second, parallel step of manually identifying relevant literature through other known sources. Table 2 describes the general inclusion and exclusion criteria that were applied to the scoping literature review.

**Table 1:** Scoping literature review search strategies

Search No	Search terms
1	Smoke OR Smoking or Tobacco OR Nicotine OR E-cigarette OR Vaping OR Snus OR Snuff OR Smokeless OR Smoking Cessation OR Waterpipe OR Hookah OR Novel tobacco product OR Modified risk tobacco product OR Reduced risk tobacco product
2	Symptom* OR Impact OR Burden OR Effect OR Quality of life OR QOL OR Well-being OR Lived experience OR ICF OR Environment OR Benefit OR Functioning OR Stress OR Activity of Daily Living or ADL OR Benefit OR Mental Health or Depression OR Anxiety OR Health status OR Cognition OR Concentration OR Memory OR Mobility OR Physical functioning OR Pain OR Discomfort OR Self-care OR Hygiene OR Getting along OR Social support OR Social functioning OR Stigma OR Role functioning OR School OR Work OR Productivity OR Leisure OR Volunteer OR Disability OR Health OR Self-esteem OR Self-confidence OR Self-efficacy
3	Qualitative OR Interpretive phenomenological analysis OR IPA OR Thematic analysis OR Grounded theory OR Content analysis OR Discourse OR Interviews OR Focus groups OR Ethnograph*
4	Quantitative OR Measure OR Questionnaire OR Patient reported outcome OR Health outcomes OR Instrument OR Diary OR Outcome OR Scale OR Survey OR Rating scale OR Linear scale OR Visual analogue scale OR VAS OR Index OR Outcome assessment OR Clinical outcome assessment OR ICF OR International Classification OR International Classification of Functioning OR WHODAS
Qualitative search strategy	1 AND 2 AND 3
Quantitative Search Strategy	1 AND 2 AND 4

**Table 2:** Inclusion and exclusion criteria applied to the scoping literature review

Inclusion criteria	Exclusion criteria
Human subjects	Animal or in vitro cell studies
Original sources of new data	Non-original sources of data
Investigate oral exposure to tobacco or nicotine products	Studies involving non-oral exposure to tobacco or nicotine products
Report at least one qualitative or quantitative positive or negative health impact or outcome related to exposure to tobacco or nicotine products	Not reporting health impact or outcomes related to exposure to tobacco or nicotine products
English language publications	Studies conducted in populations involving patients with terminal disease
	Non-English language publications
	Systematic reviews or meta-analyses

After the initial rEM exercise, two reviewers (EC, SG) further manually screened the titles and abstracts of the articles identified through the automated rEM exercise against the inclusion and exclusion criteria. Finally, the selected publications underwent a full screening by two reviewers (VL and DF) for determining their relevance to the research questions for data extraction and one of the co-authors (LA-W) cross-checked the screening and resolved differences in opinion among the reviewers.

The World Health Organization (WHO) International Classification of Functioning, Disability and Health (ICF) [28] framework and the revised Wilson and Cleary [29, 30] model were used as a guide to broadly inform categories for data extraction from the literature on TNP use and health and functioning. These established models enable the conceptualization and description of health status and functioning (the combination of which is often referred to as health-related quality of life) [31, 32], and related outcomes and determinants. To complement and refine this and to ensure relevance to those who use TNPs, the data extracted from the literature was also grouped and labeled based on the contents of the literature reviewed.

The elements extracted from the selected papers were as follows:

- Author, citation details, and publication type
- Objectives and/or research questions
- Sample type, size, and principle demographics
- Type(s) of TNP and definitions of levels of consumption
- Methodology, questionnaires, and statistical methods used
- Main results
- Results grouped in broad categories: Health Signs and Symptoms; General Health Perceptions; Quality of Life, Health-Related Quality of Life, and Functional Status; Individual Characteristics; Environmental and Social Characteristics; Biomarkers and Biological Endpoints.

### Reanalysis of focus groups/in-depth interviews

The objective of the secondary analyses of existing qualitative data in people who use TNPs was to inform the drafting of the initial conceptual framework, as well as interview guides for planned concept elicitation qualitative studies to identify concepts and develop items to detect what is relevant to measure in this context. Two sets of qualitative data containing information related to health and functioning were reanalyzed and participants had consented for their data to be used in future studies. The first was from 29 focus groups (total number of participants *n* = 229) that were originally designed to discuss perceived risk, appeal, and intent to use TNPs [33, 34]. The focus groups—stratified by smoking status—were conducted in the United States (US; *n* = 12), Japan (*n* = 4), Italy (*n* = 4), and the United Kingdom (UK; *n* = 9) between December 2012 and August 2013. The second dataset included 40 in-depth interviews conducted in North Carolina, USA, with people who use TNPs, to discuss issues centered on perceived dependence on TNPs [35]. While 21 interviewees were people who were poly-TNPs users, 19 were people who were exclusive users of one of the following types of TNPs: cigarettes (*n* = 5), smokeless tobacco (*n* = 5), e-cigarettes (*n* = 5), or another type of TNP (pipes, waterpipes, or nicotine replacement therapy [NRT] products; *n* = 4). These interviews were conducted in August 2017. The demographics of both data sets are presented in Table 3. For reanalyzing the data, an initial codebook guided by the literature review data extraction categories was developed; however, new codes were created to complement these categories based on the thematic content analysis of the transcripts. The qualitative analysis software Quirkos [36] was used for the reanalysis.

**Table 3:** Overview of the sample demographics in the qualitative studies for assessing perceived risk and dependence

Variables	Focus groups—Perceived risk* [34] (*n* = 229)	Focus groups—Dependence** [35]	
		Individuals who were exclusive users of TNPs (*n* = 19)	Individuals who were poly-users of TNPs (*n* = 21)
*Sex*			
Male, *n* (%)	109 (47.6)	12 (63.2)	13 (61.9)
Female, *n* (%)	120 (52.4)	7 (36.8)	8 (38.1)
*Age (years), mean ± SD*	39.7 ± 12.7	38.0 ± 14.95	46.0 ± 11.06
18–25 years, *n* (%)	34 (14.8)		
18–34 years, *n* (%)		7 (36.8)	7 (33.3)
26–50 years, *n* (%)	136 (59.4)		
35–49 years, *n* (%)		8 (42.1)	10 (47.6)
51–65 years, *n* (%)	59 (25.8)		
50 years or more, *n* (%)		4 (21.1)	4 (19.1)
*Race, n (%)*			
Caucasian		12 (63.1)	8 (38.1)
African-American		4 (21.1)	8 (38.1)
Other		3 (15.8)	5 (23.8)
*Education level, n (%)*			
High school or lower	68 (29.7)	6 (31.6)	8 (38.1)
Some college or college degree	142 (62.0)	5 (26.3)	7 (33.3)
Bachelor degree or beyond	19 (8.3)	8 (42.1)	6 (23.8)
*TNP use status, n (%)*			
Adult who use TNPs		19 (100.0)	21 (100.0)
Cigarettes		5 (12.5)	17 (81.0)
Cigars/cigarillos		4 (10.0)	9 (42.9)
E-cigarettes		5 (12.5)	13 (61.9)
Smokeless tobacco		5 (12.5)	10 (47.6)
Others (pipe, waterpipe, and NRTs)		0 (0.0)	4 (10.0)
Adults who smoke and with no intention to quit	71 (31.0)		
Adults who smoke and motivated to quit	39 (17.0)		
Adult who used to smoke	62 (27.1)		
Adult who never smoked	57 (24.9)		

NRT: nicotine replacement therapy; SD: standard deviation

^*^Nine focus groups conducted in London (*n* = 3), Birmingham (*n* = 3), and Glasgow (*n* = 3); four focus groups conducted in Rome and Tokyo; twelve focus groups conducted in Atlanta (*n* = 4), Los Angeles (*n* = 4), and Philadelphia (*n* = 4)

^**^In-depth interviews conducted in North Carolina, US. The sample was recruited to have equal numbers of people who use a single TNP (e.g., balanced across cigarettes, cigar/cigarillos, e-cigarettes, smokeless tobacco, and other TNPs) and people who were poly-users of TNPs

### Expert panel review

An expert panel consisting of five key opinion leaders (KOL) and five technical consultants was convened in August 28, 2018, in Neuchâtel, Switzerland. The KOLs were subject matter experts in the fields of nicotine and smoking cessation (*n* = 1), Patients Reported Outcomes (PRO) evaluation and scale development (*n* = 3), and health economics (*n* = 1). The consultants were experts on nicotine dependence (*n* = 1), psychometric validation (*n* = 2), market research (*n* = 1), and PRO development and validation (*n* = 1). The meeting followed an agenda and semi-structured discussion guide to facilitate conversations. First, the panel was presented with the principles underlying the tobacco harm reduction assessment strategy [4]. This session was followed by an open elicitation phase, during which two experienced moderators asked the panel to identify and discuss concepts related to health and functioning in people who use TNPs that different stakeholders might find important. Then, the panel was asked to review and respond to the concepts identified in the literature review and in the qualitative research reanalysis. These findings were discussed in depth to arrive at a consolidated preliminary conceptual framework. Each concept was presented, and the experts were asked to rank and agree on concepts to be included and how the concepts should be grouped by domains in the framework. In generating the framework, the project team and expert panel considered the themes and concepts identified under each of the categories from the scoping literature review, specific concepts from the secondary analyses of the qualitative data, and the expert panel meeting. The authors then synthesized and re-organized concepts emerging from the different preparatory phase activities under main health and functioning and conceptually-related domains. The participants also provided their input on the best strategies for planned qualitative studies to inform and support the development and validity of the proposed health and functioning measure.

## Results

### Scoping literature review

**Fig. 1:**
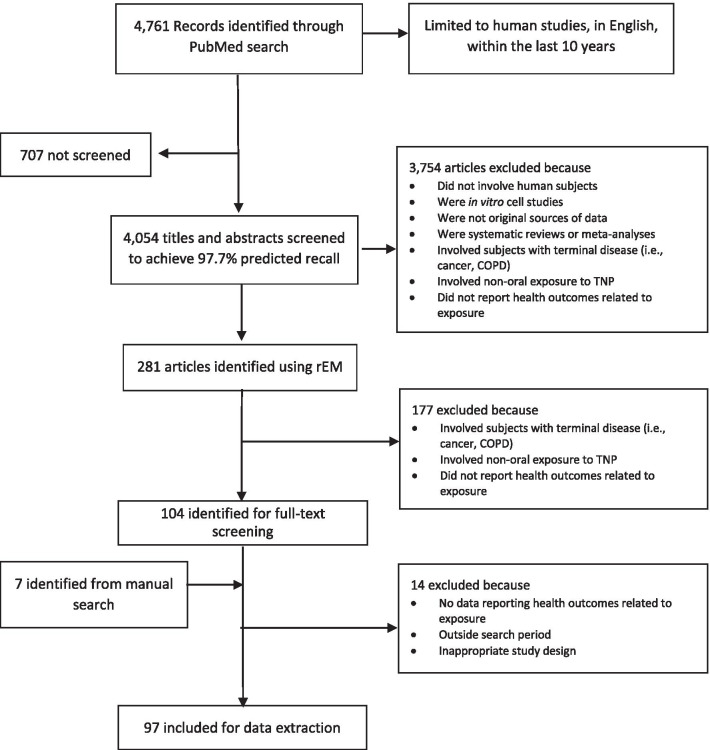
Flow diagram Sciome’s rapid Evidence Mapping (rEM) and manual screening processes of the scoping literature review

The literature search identified 4761 articles. Figure 1 (flow diagram) depicts the results of the search and screening process. Titles and abstracts were screened by the rEM exercise until the machine learning algorithms predicted 97.7% relevant references; thus, 707 abstracts were not screened. After applying the inclusion/exclusion criteria to the remaining 4,054 abstracts, 281 were identified as part of the rEM exercise. After additional manual screening and review of the abstracts and articles against the inclusion/exclusion criteria, 90 full-text articles were included for data extraction [20, 37–125]. Seven additional full-text articles were also included on the basis of a manual search [126–132]. Findings are summarized in Table 4 and a detailed description and data extracted from all the articles from the literature review is presented in Additional File 1.

**Table 4:** Summary of categories and concepts identified from the scoping literature review (97 articles reviewed)

Health signs and symptoms *n* = 56 articles (58%)	General health perceptions *n* = 18 articles (18%)	Quality of life (QoL)/Health-related Quality of Life (HRQoL)/Functional Status *n* = 9 articles (9%)	Individual characteristics *n* = 8 articles (8%)	Environmental characteristics *n* = 11 articles (11%)	Biomarkers and biological endpoints *n* = 12 articles (12%)
*Mental health and cognitive functioning* *n* = 28 articles [45, 50, 51, 54, 56, 57, 60, 61, 65, 66, 72–74, 80, 83, 91, 94, 96, 101, 105, 108, 111, 117, 124, 126, 127, 129, 130] *Oral health* *n* = 4 articles [53, 64, 90, 110] *Pain and physical trauma* *n* = 6 articles [55, 62, 77, 81, 116, 121] *Respiratory, cardiovascular and inflammatory conditions* *n* = 5 articles [59, 63, 68, 92, 125] *Other health conditions (including insomnia, liver disease, eye health, hearing loss)* *n* = 5 articles [46, 48, 85, 122, 123] *Smoking cessation-related health signs and symptoms (health benefits, dependence, addiction, withdrawal symptoms)* *n* = 8 articles [41–43, 49, 58, 103, 112, 115]	*General health perceptions and cigarettes* *n* = 9 articles [41, 52, 58, 67, 74, 78, 86, 88, 103] *Health perceptions related to e-cigarettes and other TNPs* *n* = 9 articles [52, 69, 76, 79, 97, 100, 106, 107, 118];	*Smoking and otherwise healthy individuals* *n* = 5 articles [40, 67, 73, 78, 104] *Smoking and comorbid health conditions* *n* = 1 article [77] *Nicotine reduction, smoking cessation, and quality of life* *n* = 3 articles [20, 75, 102]	*General observations (demographics, smoking and TNP use patterns)* *n* = 4 articles [37, 78, 93, 120] *Smoking cessation (intention to quit)* *n* = 1 article [70] *E-cigarettes (use patterns)* *n* = 3 articles [39, 58, 82]	*Initiating/continuing to smoke* *n* = 3 articles [89, 96, 97] *Social functioning and stigma* *n* = 5 articles [48, 75, 90, 95, 109] *Family and peer influence and Second-hand smoke* *n* = 2 articles [99, 103] *Regulations and policies regarding public smoking and tobacco control and bans* *n* = 1 article [69]	*Cardiovascular risk inflammation and physiological stress* *n* = 5 articles [38, 71, 87, 98, 114] *Depression, stress and anxiety* *n* = 2 articles [44, 92] *Internal microbiome and risk of infection* *n* = 1 article [47] *Oral health* *n* = 1 article [64] *Respiratory function* *n* = 1 article [119]; *Renal function* *n* = 1 article [84] *Metabolic function* *n* = 1 article [113]

Some articles are included in more than one category, making the total greater than 100%

Fifty-six publications (56/97; 58%) presented data related to *health signs and symptoms*. These are grouped under five core areas: *mental health and cognitive functioning* (28/97; 29%); *pain and physical trauma* (6/97; 6%); *respiratory, cardiovascular and inflammatory conditions* (5/97; 5%); *“other” health conditions*, which included insomnia, liver disease, eye health, and hearing loss (5/97; 5%); and *oral health* (4/97; 4%). There were also eight publications related to the effects of *smoking cessation* on health signs and symptoms, mostly benefits of cessation but also including perceived dependence, addiction, and withdrawal symptoms (8/97; 8%). Overall, the burden and impact of cigarette smoking on both physical and mental health symptoms was negative and generally worse among people who smoke relative to those who do not smoke. On the other hand, quitting smoking was accompanied by improvements in general physical health and psychological wellbeing. However, in spite of the positive impact of quitting smoking, loss of moments of pleasure, struggle to manage stress, the social aspects of smoking, and withdrawal symptoms were seen as barriers to quitting.

The *general health perceptions* of various adults who use TNPs were reported in 18 of the 97 articles (18%), with 9 of them detailing the general health perceptions related to cigarettes and 9 being related to e-cigarettes and other TNPs. Perceptions were determined by questionnaires and focus groups for evaluating the health impacts, fear of diseases, harm to others and self, social impacts (both positive [e.g., inclusion and looking “cool”] and negative [e.g., stigma and exclusion]), and other reasons for taking up or considering/attempting smoking cessation.

*Quality of life, health-related quality of life, and functional status* was studied in 9 of the 97 included articles (9%). These studies mostly demonstrated with generic and specific QoL, HRQoL, or functional status questionnaires that cigarette smoking was associated with a worse quality of life and that smoking cessation often resulted in an improved quality of life. However, in some cases, the use of TNPs also reportedly enabled individuals to manage their levels of anxiety and improve some aspects of social engagement and functional status.

*Individual, environmental and social characteristics* were found to influence the decision to smoke and/or consider or attempt to quit smoking or switching to other TNPs, as reported in 8 (8%) and 11 (11%) of 97 publications, respectively. Some key characteristics and determinants of smoking behavior included low socioeconomic status, male sex, living alone, family, and close social environment, societal stigma, and local regulations.

Finally, 12 of the 97 publications (12%) were related to studies on *biomarkers and biological endpoints* in people who use TNPs and showed that smoking cigarettes negatively influenced cardiovascular, respiratory, oral, renal, stress, metabolic, and inflammatory-related biomarkers and physiological assessments.

### Reanalysis of focus groups/in-depth interviews

The themes from this reanalysis are summarized below and organized on the basis of the narrative of the participants of their experiences.

#### Perceived negative impact of smoking

Other than health, the biggest and most salient reported negative impact of smoking was the perceived lack of control related to addiction and emotional health and wellbeing. Participants reported feeling that cigarette smoking was running their lives or “holding them hostage.” They reported that this perceived lack of a sense of control or willpower often led to feelings of weakness or a feeling that they were a “slave” to cigarettes. Many respondents reported smoking even when they did not necessarily want to and experiencing feelings of obsession and craving.

Perceived lack of control and addiction were also related to the activities of the participants throughout the day. People who smoke often reported altering their activities to smoke because of patterns of behavior or routine and the experienced need for a smoke. They reported that the “need for a smoke” sensation would cause them to leave work or social events early, not attend events if smoking was not allowed, interrupt what they were doing to smoke, and get up in the middle of the night.

Fear of withdrawal symptoms, with a strong emphasis on mental/emotional health, was also prominent among reported negative impacts of smoking. This fear was often reported as limiting the willingness of individuals to try to quit smoking or facilitating a return to prior smoking behavior. Individuals reported fearing the following symptoms they associated with withdrawal: mood swings and irritability, violent or aggressive behavior, inability to concentrate, anxiety, anger, and weight gain.

#### Perceived benefits of smoking

Several perceived benefits were identified that keep individuals smoking or using cigarettes. These included perceptions of enhanced cognitive functioning, relaxation, a way to take a break, use as a coping strategy, a social function, a weight management tool, the perception that it feels good, and being part of one’s identity. It is also important to note that the perceived benefits of smoking often outweighed the risks and the feeling of lack of control in the participant discussions. Even people who used to smoke noted they missed the relaxation and breaks they associated with smoking.

#### Recognition of symptoms/diseases related to smoking

Table 5 summarizes the negative symptoms and diseases related to smoking recognized by participants in both the focus groups and interviews. These were mostly related to six main body systems (cardiovascular, digestive, oral, neurological, reproductive, and respiratory).

**Table 5:** Perceived symptoms/diseases related to smoking identified from secondary analysis of qualitative studies

Body system	Symptoms/diseases
Cardiovascular	Poor blood circulation, numbness, high blood pressure, pressure in chest, high cholesterol, myocardial infarction, stroke
Digestive	Gastritis, heartburn
Neurological	Depression, jittery, dizziness, giddy, “feel different,” automatic hand movement
Oral	Sore/dry throat, bad breath, loss of sense of taste, yellow teeth, gum disease
Reproductive	Erectile dysfunction, impact on pregnancy
Respiratory	Longer time to recover from a cold, quicker to get a cold, cough, pain in the lungs, asthma, chronic obstructive pulmonary disease (COPD), wheezing, trouble breathing/slower breathing, breathlessness or shortness of breath

#### Impacts on physical functioning

The participants noted how smoking impacts their physical functioning. In particular, they noted how their exercise capacity during running, playing sports, walking upstairs, and general physical activity was diminished. They also reported reduced stamina and endurance, decreased physical strength, and feeling tired more easily.

#### Effects on emotional health

The participants also described how smoking impacts their emotional health and wellbeing. People who smoke reported feelings of shame, guilt, weakness, and a lack of control or powerlessness. They also reported feelings of depression and anxiety associated with worry about health risks. Furthermore, the participants indicated that they experienced a fear of going to places where they could not smoke, being a bad role model for their children, and (in case of people who used to smoke) going back to smoking.

#### Positive and negative social impacts

Smoking was perceived to have both negative and positive impacts on the social lives of participants. Smoking impacted life negatively when it was not allowed in certain environments, such as in homes, at work, and in cars and airplanes. Stigma was also associated with smoking in an environment where peers and family members do not smoke, but it was also seen as a source of group identity within social networks that had a higher prevalence of smoking behaviors. Participants reported that smoking had some positive impacts on their social interaction, because it facilitated work breaks and increased communication with peers.

#### Reasons people decided to try to quit

Throughout the focus groups and interviews, individuals identified several reasons why they tried to quit smoking. These included: health, diagnosis of cancer (self, family, or friend), gum disease, pregnancy, hospital stay, worry that it will “kill me,” dislike of taste or odor, social reasons, change in surroundings (fewer smoking spaces), and price.

#### Reasons people do not like alternatives to cigarettes

The participants’ reasons for not liking alternatives to cigarettes (i.e., less harmful TNPs/RRPs) included perceptions that the alternatives did not work (i.e., the participants still had cravings and experienced withdrawal symptoms), made them feel or get ill (nausea and vomiting), were not “the same” as cigarettes in terms of the ritual, taste, or “feeling,” or were inconvenient/too big to carry.

### Expert panel review

The conclusions of the expert panel widely supported the findings of the literature review and the input from the reanalyzed focus groups and interviews. Some of the experts working in field of tobacco and nicotine provided additional insights based on their extensive experience with people who use TNPs; they highlighted the importance of the enjoyment of smoking for people who find it difficult to quit, the positive immediate benefits of quitting, and the smoking-related biomarkers that might be on a causal pathway between switching and changes in health and functioning status.

The following main areas were discussed and agreed during the meeting: (1) utility of use, referring to the perceived satisfaction and enjoyment of smoking (e.g., craving relief, weight control, and social affiliation); (2) signs and symptoms of withdrawal (e.g., anxiety, depression, and anger) and the positive immediate physical health effects of quitting smoking (e.g., better general and oral hygiene, less coughing, and improved exercise capacity); (3) functioning, including cognitive, physical, sexual, social, emotional, and role functioning; (4) worry associated with smoking and smoking-related diseases; (5) general health perceptions and quality of life; (6) association with smoking-related biomarkers that could be on the causal pathway between switching and changes in health and functioning; and (7) TNP use patterns and maintenance of switching to RRPs.

### Generation of the preliminary conceptual framework

**Fig. 2:**
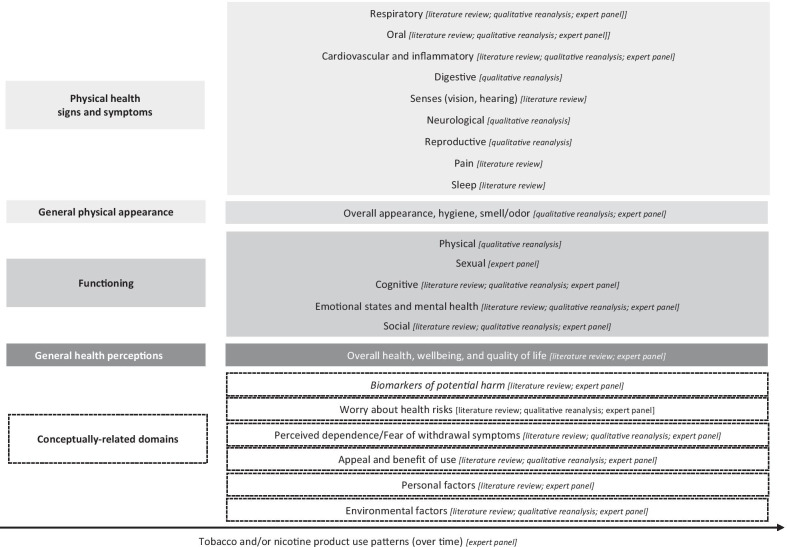
Health and functioning conceptual framework related to tobacco and/or nicotine product use from the preparatory phase research findings

Triangulation of the findings from the literature review, qualitative input from people who use TNPs, and expert panel feedback helped generate a preliminary descriptive conceptual framework that includes the health and functioning and conceptually-related domains impacted by TNP use (Fig. 2).

Four domains related to the future health and functioning measurement model for TNP use are indicated in grey rectangular boxes and include (moving down from proximal to distal parameters) physical health symptoms (e.g., oral and respiratory symptoms), general physical condition (e.g., appearance and hygiene), functioning (physical, sexual, cognitive, emotional, and social functioning), and general health perceptions, which will be the most distal measure of health and functioning. The preparatory phase research also identified six conceptually-related domains (in dashed rectangular boxes), which are not direct indicators of health status but might influence the impact of TNP use on health and functioning. These include attitudinal variables (worry about the health risks of using TNPs and perceived dependence/fear of withdrawal symptoms associated with quitting smoking), and utilitarian ones (perceived appeal, satisfaction, and benefits of TNP use). In addition, personal factors (e.g., sociodemographic) and environmental factors (e.g., peer/family influence, policies and regulations and sociocultural context) are also reflected in the conceptual framework as indirect indicators of health and functioning.

The framework further indicates that specific behavioral indicators (i.e., TNP use patterns over time) might influence any impact of TNP use on health and functioning. Whilst other causal and reciprocal relationships and hierarchies might exist within the domains, these are not explicitly characterized in this initial draft of the framework and will have to be tested with further empirical data. Finally, identified biomarkers of potential harm (in italics and dashed box) are also integrated in this conceptual framework as part of the conceptually-related domains, because they are on a causal pathway between TNP use and changes in health and functioning [133, 134]. Biomarkers are not part of the measurement model that will be considered for a new self-report measure; however, because they are the most proximal parameters to health and functioning, they will be assessed independently as appropriate endpoints by objective clinical or biological analyses.

## Discussion

Triangulation of published literature, reanalysis of qualitative data, and expert opinion helped develop the presented preliminary conceptual framework as the foundation for a new measure to assess the impact of TNPs on self-reported health and functioning. This is essential for identifying relevant concepts and understanding what is important to measure in people who use TNPs. The findings reveal the importance of not only the perceived impacts of TNP use on physical health and physical functioning, but also on aspects of mental health and social interactions and functioning, and general perceptions of health and health-related quality of life.

For the literature review, the WHO ICF [28] and Wilson and Cleary model [29, 30] served as useful guides to develop categories for data abstraction. The scoping literature review yielded 97 articles on TNP use and the relationship to health, perceptions of health, social and individual functioning, and quality of life. Overall, most studies had focused on the known negative effects of cigarette smoking (e.g., mental, respiratory, and oral health) and the rationale and motivation to quit smoking. The WHO ICF and Wilson and Clearly models were not always sufficient for identifying the breadth of relevant concepts, especially from the perspective of TNP use. Development of new codes for the reanalysis of existing qualitative data allowed for the development, extension, and exploration of the topic and provided valuable insights reported in the qualitative data reanalysis, such as the perceived benefits/satisfaction from cigarette smoking, and the rationale for quitting smoking or switching to an RRP. The findings show how this manner of secondary analysis can be valuable in health-related fields where the topic is broad and an existing body of knowledge can contribute by offering a different perspective [135].

The presentation of the preliminary conceptual framework from this preparatory phase is specific to TNP use and marks a slight departure from the established norms and characterization of the variables typically observed in existing generic health and functioning and health-related quality of life models, such as the WHO ICF and Wilson and Clearly models. Notably, specific hypothesized relationships and the hierarchy between domains are not explicitly characterized in the current draft of the framework. The framework provided an exploratory representation of the current findings to reflect a measurement instrument in people who use TNPs that would ideally be able to assess and demonstrate improvements in self-reported health and functioning status, stability of perceived positive aspects of using TNPs, and no worsening in key areas of physical and emotional health and functioning upon switching to RRPs. Nevertheless, the framework could still undergo further refinement to support the development and validation of a new measure and to further characterize and test the relationships and hierarchies between domains.

This work is not without limitations. For the scoping literature review, among the reviewed articles, not many reported on the use of e-cigarettes and other alternative tobacco or nicotine-delivery devices, because most studies had focused exclusively on cigarettes. It is possible that concepts associated with health and functioning that are relevant to other TNPs were not identified. This is most likely the consequence of the large number of publications related to cigarette use. Some concepts might also have been missed, given the large evidence base on health and functioning-related themes and concepts. However, this was also not a systematic literature search; a scoping review is generally broader than a systematic review in terms of the former having a less-defined research question, broader inclusion and exclusion criteria, and no systematic appraisal of study quality [26]. Nevertheless, the present scoping review methodology provides a lens on the overall evidence base, and regular updates on the search—specifically related to RRPs and novel TNPs and their health and functioning impacts—could be considered for fully understanding the evolving state of the art in this context. The reanalysis of existing qualitative data also has limitations related to data fit and completeness of preexisting data [136]. The insights collected from these reanalyzed studies were originally for a different purpose several years prior to the present research, and this might not completely and accurately reflect the objectives of the new project.

Considering the findings of the current research, the development of a health and functioning measure can continue to follow the FDA’s Guidance on PRO measures. As specified within the guideline, gaining input directly from the intended use populations through concept elicitation is a critical activity for ensuring content validity during the development of any new self-reported measure [137]. Continuous engagement with an expert panel can also support the refinement of the conceptual framework as well as the development of the draft and final measure.

## Conclusions

The goal of this research was to identify from varied research activities key concepts and aspects of health and functioning and related changes associated with the use of TNPs. The resulting preliminary conceptual framework provides the basis for informing future research to further understand health and functioning concepts important to measure in individual who switch to RRPs and to develop a new self-report measure to assess this from the consumers’ perspective.

## Supplementary Information

**Additional file 1.** Summary tables of results of scoping literature review

## Data Availability

All data generated or analyzed during this study are included in this published article and its supplementary information files.

## Abbreviations

ABOUT™ Toolbox: Assessment of Behavioral OUtcomes related to Tobacco and Nicotine Products Toolbox
COPD: Chronic obstructive pulmonary disease
FDA: Food and Drug Administration
HRQoL: Health-related quality of life
ICF: International Classification of Functioning, Disability and Health
MRTP: Modified risk tobacco products
NRT: Nicotine replacement therapy
PRO: Patient-Reported Outcomes
QoL: Quality of life
RRP: Reduced-risk products
rEM: Rapid Evidence Mapping
TNP: Tobacco and/or nicotine products
UK: United Kingdom
US: United States
SF-36: 36-Item Short-Form Health Survey
WHO: World Health Organization
