# Supplementary material for: Impact of tobacco and/or nicotine products on health and functioning: a scoping review and findings from the preparatory phase of the development of a new self-report measure
Source: Harm Reduct J. 2021 Jul 30;18:79. doi: 10.1186/s12954-021-00526-z (PMC8325199; doi:10.1186/s12954-021-00526-z)
Supplement: Supplementary file 1 — Additional file 1. Summary tables of results of scoping literature review [file 12954_2021_526_MOESM1_ESM.docx]

**Impact of tobacco and/or nicotine products on health and functioning: A scoping review and findings from the preparatory phase of the development of a new self-report measure**

**Additional File 1**

**Summary tables of results of scoping literature review**

Table of Tables

[Table 1: Summary Table of Publications Relating to the Category ‘Health Signs and Symptoms’ Related to Mental Health and Cognitive Functioning. 2](#_Toc77337834)

[Table 2. Summary Table of Publications Relating to the Category ‘Health Signs and Symptoms’ Related to Oral Health. 16](#_Toc77337835)

[Table 3: Summary Table of Publications Relating to the Category ‘Health Signs and Symptoms’ Related to Pain and Physical Trauma. 19](#_Toc77337836)

[Table 4: Summary Table of Publications Relating to the Category ‘Health Signs and Symptoms’ Related to Respiratory, Cardiovascular, & Inflammatory Conditions. 23](#_Toc77337837)

[Table 5: Summary Table of Publications Relating to the Category ‘Health Signs and Symptoms’ Related to ‘Other’ Health Conditions. 27](#_Toc77337838)

[Table 6: Summary Table of Publications Relating to the Category ‘Health Signs and Symptoms’ Related to Smoking Cessation. 31](#_Toc77337839)

[Table 7: Summary Table of Publications Relating to the Category ‘General Health Perceptions.’ 38](#_Toc77337840)

[Table 8: Summary Table of Publications Relating to the Category ‘Quality of Life (QoL), Health-Related Quality of Life (HRQoL), and Functional Status.’ 48](#_Toc77337841)

[Table 9: Summary Table of Publications Relating to the Category ‘Individual Characteristics.’ 52](#_Toc77337842)

[Table 10: Summary Table of Publications Relating to the Category ‘Environmental and Social Characteristics.’ 54](#_Toc77337843)

[Table 11: Summary Table of Publications Relating to the Category ‘Biomarkers and Biological Endpoints’ 56](#_Toc77337844)

Table 1: Summary Table of Publications Relating to the Category ‘Health Signs and Symptoms’ Related to Mental Health and Cognitive Functioning.

| First author, year | No. of subjects in the study | Country | Outcomes / concepts | Tools | Tobacco and/or nicotine product studied | Definition and stratification of smoking level | Results | Positive effects | Negative effects |
| --- | --- | --- | --- | --- | --- | --- | --- | --- | --- |
| Mental health and Cognitive Functioning | | | | | | | | | |
| Brody *et al* 2009 (Brody et al., 2009) | 56 | USA | Mental health; major depressive disorder (MDD) and smoking status | Hamilton Depression Rating Scale (HAM-D); Hamilton Anxiety Rating Scale (HAM-A) scores; Urge to Smoke (UTS) Scale; Spielberger State–Trait Anxiety Inventory. | Cigarettes | Number of inhalations. | MDD+ smokers had greater smoking-induced dopamine (DA) release than MDD^-^ smokers. Higher depression/anxiety levels were associated with greater smoking-induced DA release. |  | These findings support the theory that MDD+ smokers have DA system dysfunction, including heightened smoking-induced DA release. |
| Caldirola *et al* 2013 (Caldirola et al., 2013) | 100 | Italy | Mental health; Bipolar disorder (BPD) major depressive disorder (MDD) and nicotine dependence and cognitive functioning | FTND; Neuro-psychological assessment: Novelli's Story Recall Test (NSRT).  Attention Matrices: Rey–Osterrieth Complex Figure Copy Test (ROCF -C); Rey–Osterrieth Complex Figure Recall Test (ROCF-R); phonemic fluency test (PFT), semantic fluency test (SFT); Token test.  Psychometric scales: 17-item Hamilton Depression rating scale (HDRS); 18-item brief psychiatric rating scale (BPRS). | Cigarettes | Regular smokers: every day for continual four weeks, and without quitting for longer than 3 months in last 2 years. | At the beginning of hospitalization smokers showed significantly better performance in verbal memory, language fluency and working memory than non-smokers. At the end all saw an improvement in several cognitive domains but smokers maintained significantly better performance in verbal memory, language fluency and working memory. | Smokers had a significantly better performance in verbal memory and working memory domains than non-smokers.  Smokers had better performance on NSRT, PFT, and SFT than non-smokers. | BPD showed significantly worse performance on the SFT than patients with MDD. |
| Caldirola *et al* 2016 (Caldirola, Cavedini, Riva, Di Chiaro, & Perna, 2016) | 40 | Italy | Mental health; obsessive –compulsion disorder (OCD) and cognitive functioning | DSM-IV-TR criteria; Cambridge automated neuro-psychological test battery (CANTAB). | Cigarettes | Smokers: actively and continuously smoking cigarettes daily for at least 4 weeks, with no periods of non-smoking for over 3 months in the previous 2 years. | The performance of smokers and non-smokers did not differ significantly in any cognitive subdomain. The smoking duration was significantly associated with poorer visuo-spatial working memory performance (p=0.001). |  | Results did not show a benefit of smoking on cognitive function in this cohort of subjects diagnosed with OCD. |
| Depp *et al* 2015 (Depp et al., 2015) | 363 | USA | Mental health; bipolar disorder (BPD); schizophrenia and cognitive functioning | PANSS  BDI | Cigarettes | Smoking years. | Current smokers had worse composite cognitive functioning and poorer functional outcome than past or never smokers. There were no significant differences between never and former smokers, and these effects were evident in both bipolar disorder and schizophrenia. |  | In both bipolar disorder and schizophrenia, never smokers had less severe PANSS positive syndrome scale scores.  Ex-smokers with bipolar disorder had lower PANSS negative syndrome scale scores. It is worth noting that the average, adaptive functioning was significantly associated with smoking history (F2, 754=5.3, P = 0.005), with post hoc tests indicating poorer functioning among current smokers, persisting after adjustment for demographic variables (F2, 690 = 3.6, P = 0.025). Symptom severity of BDI and PANSS scores were in the mild range in both of the diagnostic groups. |
| Doiron *et al* 2017 (Doiron, Dupré, Langlois, Provencher, & Simard, 2017) | 139 | Canada | Cognitive function; diagnosis of Parkinson’s disease | Global cognitive function was assessed with the Mini-Mental State Examination (MMSE); smoking history was investigated as part of a standard clinical interview. | Cigarettes | Subjects who had ever smoked or were current smokers. | Smoking history was associated to global cognitive impairment in Parkinson’s disease even in patients who had quit smoking. | Benefit of cessation: In former smokers, the number of years since quitting had no effect on global cognition, with no significant difference between patients who had quit smoking more than 10 years ago and those who had quit less than 10 years ago. | The ever-smokers performed significantly worse on the MMSE than the never-smokers. |
| Gonzalez *et al* 2008 (Gonzalez, Zvolensky, Vujanovic, Leyro, & Marshall, 2008) | 189 | USA | Mental health; smoking cessation | Mini-Mental State Examination (MMSE); DSM-IV Axis I disorders; SHQ; FTQ; Alcohol use disorders identification test; marijuana smoking history questionnaire; Positive affect negative affect scale; Anxiety sensitivity index; difficulties in emotion regulation scale; reasons for smoking; barriers to cessation scale. | Cigarettes | Cigarettes per day. | Results indicated that anxiety sensitivity (AS) was significantly related to coping, addictive, and habitual smoking motives, as well as greater perceived barriers to quitting. |  |  |
| Heffernan *et al* 2012 (Heffernan, O'Neill, & Moss, 2012) | 69 | UK | Mental health and cognitive function | Prospective and Retrospective Memory Questionnaire (PRMQ); Real World Prospective Memory Task (RWPMT); smoking and other drug use was assessed by a Recreational Drug Use Questionnaire; The Hospital Anxiety and Depression Scale. | Cigarettes | Current and previous smokers. | The analyses revealed no significance between-group differences on self-reported prospective memory (PM); however, smokers recalled significantly fewer action–location combinations than the never-smokers and former-smokers on the objective real-world PM tasks. |  |  |
| Highland *et al* 2011 (Highland & McChargue, 2011) | 79 | USA | Coronary heart disease (CHD), mental health and behavior | Caffeine and Alcohol intake form, FTND; Beck Depression Inventory-II; Negative mood questionnaire (assesses mood evoking autobiographical events); Vividness scale; PANAS; | Cigarettes | 15 cigarettes per day for at least 2 years, carbon monoxide reading of 15ppm or greater. | African American (AA) hypertensive smokers showed greater elevations in their systolic responses compared to normotensive controls following the laboratory induced stressor. Findings revealed a potential stress-induced mechanism suggesting that AA hypertensive smokers were more vulnerable to CHD risk than their normotensive counterparts. |  | Cigarette smoking associated with an elevated risk of CHD. Smoking and hypertension were independent health risks but together appeared to have a negative, synergistic effect on mortality risk in the AA group. |
| Kahler *et al* 2009 (Kahler et al., 2009) | 1130 | USA | Mental health, nicotine dependence | Multidimensional Personality Questionnaire; Lifetime Interview of Smoking Trajectories and the Quitting Methods Questionnaire; Composite International Diagnostic Interview; Diagnostic Interview Schedule. | Cigarettes | Current, former and never smokers. | Current smokers reported being more reactive to stress, more aggressive, more alienated, and less harm avoidant than both former smokers and never-smokers, whereas former smokers and never-smokers showed similar personality profiles overall. |  | Smoking impacts on substance and behavioral dependence:  Current and former smokers had significantly higher rates of lifetime alcohol dependence, substance dependence, conduct disorder, and any externalizing or any psychiatric disorder compared to never-smokers.  Impacts on psychiatric disorders:  Current smokers also were more likely than never-smokers to have a history of major depressive disorder compared to former smokers; current smokers had higher rates of all disorders other than depression.  Compared to never-smokers and former smokers, current smokers had significantly higher scores on stress reaction, aggression and alienation compared to former and never-smokers.  Current smokers also had significantly lower scores on control compared to never-smokers. |
| Lappan *et al* 2018 (Lappan, Thorne, Long, & Hendricks, 2018) | 2939 to 4230 | USA | Psycho-logical well-being | Satisfaction with Life Scale; Life Orientation Test – Revised; National Survey of Midlife Development; Positive and Negative Affect Schedule; Ryff Measures of Psychological Well-being. | Cigarettes | The biennial Health and Retirement Study (HRS) has longitudinally surveyed a representative sample of Americans aged 50 years and older since 1992 to describe the health, psychological, financial, work, insurance, and retirement planning conditions of these adults. | A modelling exercise using cohorts from the longitudinal Health and Retirement Study showed that smoking in 2006 was associated with less psychological well-being (PWB) in 2010. Specifically, though there was no association between purpose in life in 2006 and smoking status in 2010, greater life satisfaction, optimism, and positive affect measured in 2006 were each predictive of a reduced likelihood of smoking in 2010. |  | Being a smoker in 2006 predicted lower levels of life satisfaction (standardized path coefficient = -0.25), optimism (standardized path coefficient = -0.10), positive affect (standardized path coefficient = -0.10), and purpose in life (standardized path coefficient = -0.13) in 2010. |
| Lynch *et al* 2011 (Lynch, Johnson, Kable, Carroll, & Coles, 2011) | 218 | USA | Mental health and behavior | Hospital interview | Cigarettes | Smokers vs Non-smokers  Smokers: light smokers (average of ≤14 cigarettes/day); heavy smokers (average of 15 or more cigarettes/day). | Results showed  (a) maternal smoking during pregnancy predicted maternal report of parenting stress at six months postpartum and  (b) maternal psychological symptoms mediated the relationship between smoking during pregnancy and mothers’ experience of parenting stress, but socioeconomic status did not. |  | Results suggested that children of the mothers who smoked during pregnancy and experienced more psychological problems could be at increased risk for developing behavioral problems. |
| Lyvers *et al* 2014 (Lyvers, Carlopio, Honours, & Edwards, 2014) | 183 | Australia | Mental health and behavior; smoking cessation/ reduction | Depression Anxiety Stress Scales (DASS-21); Negative Mood Regulation (NMR) scale; Frontal Systems Behavior Scale (FrSBe), FTCD; Alcohol Use Disorders Identiﬁcation Test. | Cigarettes | Current smokers who have smoked daily for at least one year; ex-smokers who had not smoked a cigarette for at least one year, and never-smokers. | Long-term abstinent ex-smokers and never-smokers did not signiﬁcantly differ on any measure, whereas current smokers indicated signiﬁcantly worse functioning on all measures compared to the other two groups.  Chronic smokers may believe that smoking alleviates stress, anxiety, or depression, but the evidence to date suggests that they would be better off by quitting. | The study provided support for the notion that quitting smoking could eventually lead to improvements in mood and general functioning (as well as additional health benefits).  The ﬁnding that ex-smokers were like never-smokers on all measures suggested that difficulties such as mood / frontal systems dysfunction tended to resolve with long term abstinence. | The ﬁnding that chronic smokers experienced emotional barriers to quitting may not reflect objective data on the benefits of smoking cessations. |
| McLeish *et al* 2009 (McLeish, Zvolensky, Del Ben, & Burke, 2009) | 51 | USA | Mental health and behavior; quality of life; nicotine dependence. | Semi-structured interview. | Cigarettes | 10 cigarettes a day and who had made a cessation attempt. | Results indicated that the interaction between asthma and smoking rate signiﬁcantly predicted anxious arousal, agoraphobic avoidance, and anticipatory anxiety. |  | The combination of higher smoking rates and higher levels of asthma was associated with greater anxious arousal. |
| Mendiondo *et al* 2010 (Mendiondo, Alexander, & Crawford, 2010) | 31428 | USA | Mental health and behavior; lung function; CV health; diabetes; nicotine dependence. | National interview survey to monitor the public on range of health topics. | Cigarettes | Former and current smokers stratified by number of cigarettes per day, lifetime, and brand type. | Compared to current non-menthol smokers, current menthol smokers were more likely to have been told that they had diabetes, asthma and had asthma at time of the survey.  Former menthol smokers, compared to former non-menthol smokers, were more likely to still have asthma and visit the emergency room due to this. |  | Compared to current non-menthol smokers, current menthol smokers were more likely to have poor mental health and wellness and reported being told more often that they had diabetes. |
| Nikcevic *et al* 2010 (Nikcevic & Spada, 2010) | 12 | USA | Mental health and behavior, weight; smoking; smoking cessation. | FTND. | Cigarettes | Regular smokers: smoking ≥10 cigarettes daily. | Results indicated that participants endorsed both positive and negative metacognitive beliefs about smoking and that the goal of smoking was to regulate negative emotion and/or to enhance cognitive functioning. | Sensations of smoking i.e. taste and inhalation of smoke were concepts that gave pleasure during smoking and used as a means to reduce stress and improve cognitive function. | Negative beliefs regarding smoking included lack of control (i.e. urge to smoke) as well as negative impact on self-appraisal and cognitive functioning.  Dependence on smoking to achieve clarity of thought was considered a risk.  Socializing had a negative impact on the amount smoked, i.e. 'external' factors rather than internal emotional states. |
| Pasco *et al* 2008 (Pasco et al., 2008) | 1043 | Australia | Mental health and behavior. | DSM-IV-TR. | Cigarettes | Smoking more than one or two cigarettes per day for at least 6 months, and recorded details of smoking included frequency and period of exposure. | Smoking was associated with increased odds for major depressive disorder. Compared with non-smokers, odds for major depressive disorder more than doubled for heavy smokers (>20 cigarettes/day). |  | Smoking was associated with MDD. Findings were suggestive of a dose-dependent association, with more than a two-fold increase in the odds of major depressive disorder for heavy smokers compared to non-smokers. |
| Poole-Di Salvo *et al* 2010 (Poole-Di Salvo, Liu, Brenner, & Weitzman, 2010) | 30,668 | USA | Behavioral and emotional patterns among children of smokers. | Columbia Impairment Scale score. | Cigarettes | Maternal smokers. | The odds of Columbia Impairment Scale score increased with increasing number of smokers in the household, even among children whose mothers did not smoke. |  | Children living with smokers were at increased risk for emotional or behavioral problems, and rates of such problems increase with increasing numbers of smokers in the household, even in the absence of maternal smoking. |
| Roth *et al* 2013 (Roth, Bengtsson, & Ohlsson, 2013) | 250 | Sweden | Mental health | GI Symptom Rating Scale (GSRS); psychological general well-being index (PGWB). | Cigarettes; nicotine gum; snuff. | Never smoked; stopped smoking; current smokers; consumers of nicotine chew or nicotine snuff. | Smoking was associated with impaired GI symptoms.  Patients with IBS only had impaired scores for the dimensions of abdominal pain and constipation compared to the whole group diagnosed with microscopic colitis.  The only lifestyle factor that affected whether the patient fulfilled IBS criteria was smoking which increased the risk for also suffering from IBS-like symptoms. |  | Smoking was associated with impaired psychological well-being and impaired GI symptoms. |
| Segarra *et al* 2011 (Segarra et al., 2011) | 41 | Spain | Mental health and behavior | Psychiatric assessment: SCID used for diagnostics; PANSS; MADRS; UKU Side effect rating scale.  Cognitive function: Continuous Performance Test-O/CPT-O; Stroop Color-Word Test Interference score/Stroop-I; Continuous Performance Test-XO/ CPT-XO. | Cigarettes | Non-smokers; current smoker (>15 cigarettes a day) | Among subjects experiencing a first psychosis, non-smoking patients showed signiﬁcant cognitive improvements over time, whereas smoking patients lost their superior baseline performance.  Smoking was not associated with fewer extrapyramidal side effects. | Smoking might improve attention and working memory to a modest extent as atypical antipsychotics could reﬂect an effort to ameliorate these cognitive dysfunctions previous to treatment instauration.  Between group comparison: smokers showed signiﬁcantly faster reaction times with a lower percentage of errors than non-smokers in the Stroop-I task. They also showed a signiﬁcantly lower percentage of omission errors in the CPT-XO task. | Smokers did not obtain any cognitive beneﬁt after initiation of treatment and symptoms worsened over the ﬁrst year. |
| Talati *et al* 2013 (Talati et al., 2013) | 4326 | USA | Mental health, nicotine dependence | FTND; psychiatric diagnoses (drug and alcohol dependence, major depression, and generalized anxiety disorder) using the Composite International Diagnostic Interview-Short Form; Personality traits (neuroticism and extraversion) with the Eysenck Personality Questionnaire. | Cigarettes | Three smoking levels were used: Lifetime dependent smoker, never dependent smoker, never smoker. | Lifetime prevalence of smoking decreased across the seven cohorts over the past seven decades. Associations between smoking and drug dependence, generalized anxiety, and neuroticism, as well as total psychiatric comorbidity, were greater in more recent cohorts, with greatest increases contributed by nicotine-dependent smokers. |  | More recent generations included fewer persons who smoked, but their smoking was associated with greater psychiatric morbidity. |
| Taylor *et al* 2014 (Taylor et al., 2014) | 26 studies | NA | Smoking cessation, Mental health | State Trait Anxiety Inventory Hospital Anxiety and Depression Scale Brief Symptom Inventory. | Cigarettes | Smokers in the general population. | Smoking cessation was associated with reduced depression, anxiety, and stress and improved positive mood and quality of life compared with continuing to smoke. The effects were as large for those with psychiatric disorders as those without. |  | Compared to continuing to smoke, quitting smoking was associated with a significant decrease in anxiety from baseline. |
| Zorlu *et a*l 2017 (Zorlu et al., 2017) | 50 | Turkey | Mental health and behavior; brain structure. | DSM-IV (SCID); Hamilton Depression rating scale (HDRS-17); FTND. | Cigarettes | Smokers vs  non-smokers. | The smoking group showed significant cortical thinning in comparison to the non-smoker MDD group in select temporal and frontal regions. |  | Smoker vs non-smoker: Left hemisphere non-smoker MDD group showed significantly increased thickness in two clusters (i.e. middle temporal cortex and post central gyrus).  Surface area: Non-smoker MDD group showed significant increased area in one cluster (lateral occipital cortex) of right hemisphere.  No statistically significant difference in surface area in the left hemisphere between non-smoker MDD and healthy control groups. |

Table 2. Summary Table of Publications Relating to the Category ‘Health Signs and Symptoms’ Related to Oral Health.

| First author, year | No. of subjects in the study | Country | Outcomes / concepts | Tools | Tobacco and/or nicotine product studied | Definition and stratification of smoking level | Results | Benefits reported | Harms reported |
| --- | --- | --- | --- | --- | --- | --- | --- | --- | --- |
| Oral health | | | | | | | | | |
| Croucher *et al* 2013 (Croucher, Haque, & Kassim, 2013) | 150 | UK | Oral health, pain and trauma, smoking cessation | Psychological distress~: Assessed using the 14-item culturally adaptable Hospital Anxiety and Depression Scale (HADS) questionnaire.  Proximal behavioral data: medical history, dental service utilization, and type of smokeless tobacco consumed  Prevalence of oral pain: dichotomous (yes/no) responses to an item taken from a 14-item oral pain questionnaire | Paan quid with tobacco (PQT) | Regular daily PQT chewers and with an intention to make a smokeless tobacco cessation attempt. | The prevalence of oral pain at baseline was 39%, increasing at follow-up to 73%. Thirty-eight percent reported a dental visit in the previous 12 months. While 77% had 21 teeth or more, tooth wear was common. |  | Chewing PQT led to tooth wear, attrition and abrasions.  Cessation was associated with an increase in the prevalence of oral pain. |
| Javed *et al* 2017 (Javed et al., 2017) | 94 | Saudi Arabia | Oral health | Standardized oral health related questionnaire. | Cigarettes; e-cigarettes | Cigarette smokers (CSs) (group 1), individuals exclusively vaping electronic cigarettes (group 2), and never-smokers (NSs) (group 3) | Periodontal inﬂammation and self-perceived oral symptoms were exacerbated in CSs compared to vaping individuals and NSs. | E-cigarettes reduced periodontal inflammation and improved perceived oral health compared to cigarettes, although e-cigarettes could not be considered non-hazardous. | Periodontal inﬂammation and self-perceived oral symptoms were greater among CSs than among vaping individuals and NSs. |
| Nemeth *et al* 2012 (Nemeth et al., 2012) | 23 | USA | Social integration/ stigma, aesthetics, oral health | Qualitative interviews and focus groups. | Smokeless tobacco (ST): chewing tobacco, dry and moist snuff | Smokeless tobacco (ST) users vs. non-users | Cultural standards dictated that tobacco use was a necessary rite of passage in the development of masculine identity in Appalachian Ohio. A person’s male social network was consistently cited as the primary inﬂuence on ST initiation and continued use. | Cultural beliefs played a large role in ST use among males. Smoking was perceived as a rite of passage, with users believing their acceptance into social networks was predicated on ST use. | Users were aware of some risk to use of ST, in particular related to teeth turning ‘nasty’. Males were less concerned about appearance than females but felt that females chewing tobacco was less acceptable and a turn off, i.e. they did not want to be with someone with nasty teeth. |
| Tatullo *et al* 2016 (Tatullo, Gentile, Paduano, Santacroce, & Marrelli, 2016) | 110 | Italy | Oral health, smoking cessation or reduction | Oral health assessment, plaque index (PI) and periodontal bleeding index (BI). | Cigarettes and e-cigarettes | Cigarette users (≥20 per day) that switched to e-cigarettes completely. | Clear improvements in specific oral health parameters were observed after switching to e-cigarettes. | Overall, 71% reported better oral health and 78% noted reductions in the frequency of respiratory disease. A positive response to smell and taste was also noted. |  |

Table 3: Summary Table of Publications Relating to the Category ‘Health Signs and Symptoms’ Related to Pain and Physical Trauma.

| First author, year | No. of subjects in the study | | Country | Outcomes / concepts | Tools | Tobacco and/or nicotine product studied | Definition and stratification of smoking level | Results | Benefits reported | Harms reported |
| --- | --- | --- | --- | --- | --- | --- | --- | --- | --- | --- |
| Pain and Physical Trauma | | | | | | | | | | |
| Croucher *et al* 2013 (Croucher et al., 2013) | | 150 | UK | Pain and trauma, smoking cessation | Psychological distress~: Assessed using the 14-item culturally adaptable Hospital Anxiety and Depression Scale (HADS) questionnaire.  Proximal behavioral data: medical history, dental service utilization, and type of smokeless tobacco consumed  Prevalence of oral pain: dichotomous (yes/no) responses to an item taken from a 14-item oral pain questionnaire | Paan quid with tobacco (PQT) | Regular daily PQT chewers and with an intention to make a smokeless tobacco cessation attempt. | The prevalence of oral pain at baseline was 39%, increasing at follow-up to 73%. Thirty-eight percent reported a dental visit in the previous 12 months. While 77% had 21 teeth or more, tooth wear was common. Eighty-five percent of participants were observed to have attrition and 73% abrasion. Anxiety prevalence changed from 86% to 78.7% and depression changed from 74.7% to 77.3%, comparing baseline with study completion. | Chewing PQT limited, but did not completely prevent or eradicate, oral pain.  Cessation was associated with reduced anxiety, although prevalence was still high 12 months after quitting. | Chewing PQT led to tooth wear, attrition and abrasions.  Cessation was associated with an increase in the prevalence of oral pain. |
| Ditre *et al* 2017 (Ditre, Zale, Heckman, & Hendricks, 2017) | | 75 | USA | Pain and trauma, smoking cessation | Pain and Smoking Inventory (PSI) | Cigarettes | Cigarettes per day: light smokers: <5; moderate to heavy: 5 or more per day | PSI scores were higher among smokers with chronic pain versus no pain, and were positively associated with established indices of both pain and tobacco smoking dependence. |  | PSI total scores were positively associated with pain intensity, interference with functioning and the severity of both sensory and affective components of the pain experience (all p<0.01).  Smokers who scored higher on the PSI also reported greater levels of pain catastrophizing and discomfort intolerance, as well as greater levels of pain-related emotional distress (all p<0.01). |
| Holley *et al* 2013 (Holley et al., 2013) | | 794 twin pairs (1588) | USA | Musculo-skeletal, mental health | Pain intensity rated on an 11-point scale. Depression assessed using the Patient Health Questionnaire (PHQ2); Anxiety measured using the Brief Symptom Inventory; Stress assessed using the Perceived Stress Scale. | Cigarettes | Current smokers and at least 100 cigarettes in a lifetime. | Results revealed a near 2-fold increase for chronic musculoskeletal pain in twins who smoked compared to those who did not. Twins with chronic pain also had higher anxiety and more stress.  Data suggests anxiety about pain may reinforce smoking as a tool for reducing negative affect. |  | Those with a history of chronic musculoskeletal pain were more likely to report current smoking. Those with such pain also had higher than usual pain intensity, higher perceived stress and greater anxiety, and were more likely to screen for depression. |
| Mattey *et al* 2011 (Mattey, Dawson, Healey, & Packham, 2011) | | 612 | UK | Pain and trauma; health-related quality of life; inflammation; nicotine dependence; | Bath Ankylosing Spondylitis Disease Activity Index (BASDAI) | Cigarettes | Dependence level based on smoking status and duration, average number of cigarettes smoked per day, and age at smoking cessation.  Smoking status categorized participants into current smokers, past smokers, and those who had never smoked. | Study showed a negative effect of smoking on disease activity, pain, function, and quality of life.  Increasing severity of outcome measures with increasing number of pack years suggested that cessation of smoking in this patient cohort may be beneficial to reduce pain related to inflammation. |  | BASDAI scores were higher in smokers than non-smokers.  Pain was primarily associated with current smoking rather than pack–year history.  Smoking was associated with worse functional ability.  Smoking impaired quality of life. |
| Melis *et al* 2010 (Melis et al., 2010) | | 352 | USA | Pain, oral health, nicotine dependence | Clinical charts | Cigarettes | Subjects were first divided into two groups: smokers (YS) and non-smokers (NS); then, YS were further divided into three subgroups: light smokers (LS), moderate smokers (MS), and heavy smokers (HS). | Higher intensity of temporomandibular joint pain was experienced by smokers compared to non-smokers, with increasing values with increasing levels of smoking. |  | A positive correlation was found between the number of cigarettes smoked in a day and pain intensity for the entire sample. |
| Volkman *et al* 2015 (Volkman et al., 2015) | | 406,954 | USA | Pain and trauma | Self-reported pain questionnaire | Cigarettes | Never, current, and former smokers | In military veterans, there was an association between current smoking and pain intensity. This effect was attenuated in former smokers.  In a subgroup analysis by gender, it was observed a higher pain severity for current smokers in both men and women Veterans. | Smoking cessation can help to reduce pain experienced by military veterans. | Given the impact smoking has on the health status of young veterans, smoking prevention and cessation programs are also needed in the military. Smoking cessation is a challenging behavior to change and veterans with chronic pain may experience more barriers to quitting. |
| Vujanovic *et al* 2010 (Vujanovic, Marshall-Berenz, Beckham, Bernstein, & Zvolensky, 2010) | | 63 | USA | Mental health; pain and trauma | FTND; Structured Clinical Interview for DSM-IV axis I disorder; Post-traumatic Diagnostic Scale (PDS); Smoking History Questionnaire (SHQ); Minnesota Nicotine Withdrawal Scale; Subjective Units of Distress Scale (SUDS) | Cigarettes | Daily smokers for at least the past year consuming at least 10 cigarettes per day for the prior month and had not decreased the number of cigarettes smoked per day by more than half in the preceding six months. | Post-traumatic stress symptom severity and the smoking-as-usual conditions were significantly predictive of anxiety during a cigarette withdrawal challenge. The effects of withdrawal were greatest at 3 and 4 minutes of the test, but had dissipated by minute 9. |  | In subjects with post-traumatic stress, nicotine withdrawal was associated with a rapid short-term increase in anxiety. |
| Yu *et al* 2015 (Yu et al., 2015) | | 215 | China | Pain and trauma | FTND; level of patient-controlled intravenous analgesia post-surgery (sufentanil); pain intensity was assessed using a numerical rating scale (NRS). | Cigarettes | Smokers: smoking history of >1 year and discontinued smoking for less than 1 month before surgery.  Non-smokers (NS): patients who had no history of smoking or discontinued smoking for more than 3 months.  Dependence: low-nicotine dependent (LD): FTND score <6; highly-nicotine dependent (HD): FTND score ≥6. | NRS scores and total amount of self-administered sufentanil were signiﬁcantly higher in the HD and LD groups compared to the NS group, as well as being signiﬁcantly higher in the HD group than in the LD group. The FTND scores positively correlated with the cumulative quantity of sufentanil at 24 and 48 hours after surgery. |  | Smoking appeared to alter pain sensing and processing, resulting in increased pain sensitivity. Smokers had more severe postoperative pain and required a higher cumulative dosage of sufentanil than did non-smokers. In addition, postoperative pain severity and the need for postoperative opioid self-treatment correlated with the degree of nicotine dependence in smokers. |

Table 4: Summary Table of Publications Relating to the Category ‘Health Signs and Symptoms’ Related to Respiratory, Cardiovascular, & Inflammatory Conditions.

| First author, year | No. of subjects in the study | Country | Outcomes / concepts | Tools | Tobacco and/or nicotine product studied | Definition and stratifi-cation of smoking level | Results | Benefits reported | Harms reported |
| --- | --- | --- | --- | --- | --- | --- | --- | --- | --- |
| Respiratory, Cardiovascular, & inflammatory Conditions | | | | | | | | | |
| Hawari *et al* 2017 (Hawari, Obeidat, Ghonimat, Ayub, & Dawahreh, 2017) | 138 | Jordan | Respiratory | Respiratory health and cardio-pulmonary exercise tests; American Thoracic Society and the Division of Lung Diseases (ATS-DLD-78) adult questionnaire. | Waterpipe | Length of time used | Exercise time was significantly shorter for waterpipe smokers (WPS) than non-smokers  With regards to respiratory symptoms, a significantly greater proportion of WPS (72.5%) than non-smokers (21.7%) reported any respiratory symptoms (e.g. bringing up phlegm, having shortness of breath upon exertion, cough, chest illness in the past three years that kept a participant off work, and coughing with phlegm that lasted at least three weeks).  WPS had significantly lower FEV1, FVC, PEF and TLC. |  | WPS were more likely than non-smokers to report respiratory symptoms. |
| Ichikawa *et al* 2014 (Ichikawa et al., 2014) | 26 | Japan | Cardiovascular health | Breath-hold phase- contrast cine magnetic resonance (MR) imaging of coronary sinus. | Cigarettes | Length of time; cigarettes per day; smokers Vs non-smokers | In non-smokers, myocardial blood flow (MBF) was 0.88 ± 0.19 ml/min/g at rest and significantly increased to 1.13 ± 0.26 ml/min/g during the cold  Cold pressor test (CPT; p=0.0001).  In smokers, MBF was 0.94 ± 0.26 ml/min/g at rest and 0.96 ± 0.30 ml/min/g during the CPT (P = 0.73). DMBF (MBF during the CPT–MBF at rest) was significantly reduced in smokers compared to non-smokers (0.02 ± 0.20 vs. 0.26 ± 0.18 ml/min/g, P = 0.005).  The intra-class correlation coefficient between measurements by two observers was 0.90 for DMBF. A significant reduction in MBF response to CPT was demonstrated in young smokers with PC-MRI at 1.5 T. The mean percent change of  MBF in smokers was also significantly lower than in non-smokers (3.6 ± 19.7 vs. 30.7 ± 21.3 %, p = 0.003). |  | Smoking had a negative impact on myocardial blood flow (MBF). |
| Johnson *et al* 2017 (Johnson & McLeish, 2017) | 235 | USA | Respiratory and mental health | Multi-dimensional Personality Questionnaire. | Cigarettes | Smokers with asthma and without asthma. Cigarettes per day, length of use. | After controlling for the effects of cigarettes per day, gender, race, and education, smokers with asthma reported higher levels of anxiety, sensitivity, and panic symptoms, and were at an increased risk for having a lifetime history of panic attacks (OR = 3.01) and panic disorder (OR = 2.96), compared to smokers without asthma. Further, group differences in anxiety sensitivity and panic symptoms remained even after removing participants with a lifetime history of panic attacks or panic disorder. |  | Smokers with asthma were an at-risk population for anxiety and panic. |
| Kim *et al* 2012 (J. Kim et al., 2012) | 1589 | Australia | Cardiovascular health/ disease | SF-36 | Cigarettes | Current smokers, ex-smokers, never smokers | Patients who were current smokers at the time of their stroke had poorer outcomes compared to those who had never smoked. | There were benefits to smoking cessation, with ex-smokers appearing to have a lesser risk of recurrent vascular events than current smokers. | Among those who survived the first 28 days of stroke, current smokers and ex-smokers at baseline had poorer outcome than those who had never smoked.  Current smokers also had a greater risk of recurrent events than past smokers.  Patients who smoked at the time of their stroke or had smoked before their stroke had greater risk of death or recurrent vascular events when compared to patients who were never smokers. |
| Miyatake *et al* 2011 (Miyatake, Numata, et al., 2010) | 825 | Japan | Lung function and associated disease, cardio-vascular health, non-invasive technique | Annual Health checkup | Cigarettes | Current smokers, total per day | Exercise habits were closely linked to cigarette smoking in men, and the differences in parameters at ventilatory threshold (VT) between subjects with and without cigarette smoking were attenuated after adjusting for age and exercise habits.  However, in women without exercise habits, oxygen uptake at VT in women with cigarette smoking was signiﬁcantly lower than that in women without, after adjusting for age. |  | Oxygen uptake and work rate at VT in subjects with cigarette smoking were signiﬁcantly lower than those in subjects without cigarette smoking. |
| Nunes *et al* 2012 (Nunes et al., 2012) | 155 | Brazil | Inflam-matory conditions | Nicotine dependence in accordance with DSM-IV; FTND | Cigarettes | Smokers recruited from a smoking cessation program | Depressed smokers had higher hs-CRP, IL-6, and TNF-a levels than non-depressed smokers, had worse physical health outcomes and greater work-related disability. |  | Depressed smokers were more likely to be hospitalized in the previous month than non-smokers. Also, more likely to suffer CV and respiratory disease and to have more work-related disability.  Individuals who smoke and were depressed had higher levels of inflammatory biomarkers, particularly CRP levels, TNF alpha, and IL-6, than non-depressed smokers. Levels of serum TNF-a and IL-1 were associated with increased risk of depression, cognitive impairments, and reduced responsiveness to treatment. |

Table 5: Summary Table of Publications Relating to the Category ‘Health Signs and Symptoms’ Related to ‘Other’ Health Conditions.

| First author, year | No. of subjects in the study | Country | Outcomes / concepts | Tools | Tobacco and/or nicotine product studied | Definition and stratification of smoking level | Results | Benefits reported | Harms reported |
| --- | --- | --- | --- | --- | --- | --- | --- | --- | --- |
| Other Health Conditions | | | | | | | | | |
| Brook *et al* 2012 (Brook, Rubenstone, Zhang, & Brook, 2012) | 498 | USA | Insomnia | Structured interviews | Cigarettes | Cigarette smoking was assessed using a summative index. At each wave of data collection, subjects were asked to report the number of cigarettes smoked daily: none (0), up to half a pack (1), half to one pack (2), more than one pack (3). Late quitters smoked about a pack of cigarettes a day until mean age 43 years, and quit completely by age 65 years. | Among the smoking trajectory groups, the prevalence of insomnia per group was: non-smokers: 5.9%; late quitters: 10.6%; moderate smokers: 12.5%; chronic, heavy smokers: 20.0%. There were statistically significant differences between the groups (p=0.004). |  | Compared to non-smokers, members of the heavy smoking trajectory group were more likely to report insomnia at mean age 65. |
| Brown- Johnson *et al* 2015 (Brown-Johnson et al., 2015) | 956 | USA | Social stigma, mental health | Internalized Stigma of Smoking Inventory (ISSI) | Cigarettes | Smokers: Cigarette smoking was assessed by use of a summative index. | Self-stigma was greatest among those intending to quit; felt stigma from others was highest among those experiencing stigma in other ISSI domains, namely ethnicity, mental illness, and smoking-related discrimination. Felt stigma was highest among women, Caucasians, and those with the highest educational level. |  | Stigma by the self and others was found to be a key factor in quitting smoking. |
| Mohammadi *et al* 2009 (Mohammadi, Mazhari, Mehrparvar, & Attarchi, 2009) | 504 | Iran | Hearing loss | Standardized audiometric examination | Cigarettes | Smokers | Among employees exposed to noise levels >85dBA, smoking increased the likelihood of hearing loss. Among smokers exposed to noise levels >85dBA, high-frequency hearing loss was associated with increasing pack-years of smoking. |  | Smoking accelerated hearing loss in this study. The odds ratio for hearing loss increased in parallel with the number of pack-years of smoking. |
| Zhao *et al* 2011 (Zhao, Xu, Lai, Che, & Zhou, 2012) | 181 | China | Liver disease, mental health, behavior | A battery of self-report questionnaires: smoking status and motivation; Hospital Anxiety and Depression Scale (HADS); Smoking History Questionnaire; Reasons for Smoking Questionnaires (RSQ). | Cigarettes | Smokers vs. non-smokers | ‘Anxiety’ and ‘depression’ were the primary motivation for the increase in cigarette consumption after the diagnosis of hepatitis B and associated with continued smoking after diagnosis.  Anxiety frequently coexisted with depression in smokers with hepatitis B (these types of patients were shown to have poorer outcomes and symptoms). | ‘Nicotine pleasure’ was the primary motivation before the diagnosis of hepatitis B. | Number of cigarettes per day after a diagnosis of hepatitis B was higher than before the diagnosis.  Ability to quit was adversely affected by presence of anxiety and depression. |
| Zhang *et al* 2011 (Zhang et al., 2011) | 36522 | USA | Eye health | Visual impairment was assessed in the vision module with the question, e.g., “How much difficulty, if any, do you have in recognizing a friend across the street?” and “How much difficulty, if any, do you have reading print in newspaper, magazine, recipe, menu, or numbers on the telephone?” | Cigarettes | Current smokers: at least 100 cigarettes ever and still smoked at time of interview; former smokers: ever smoked at least 100 cigarettes but not smoking at time of interview; never smoker: smoking fewer than 100 cigarettes in their lifetime and not smoking at the time of interview. | Among respondents with age-related eye diseases, the estimated prevalence of visual impairment was statistically significantly higher among current smokers (48%) than among former smokers (41%, p<0.05) and respondents who had never smoked (42%, p<0.05).  Among respondents with cataracts, the predictive margin of having visual impairment was higher among current smokers than never smokers (44% vs. 40%, p=0.03).  Among respondents with age-related macular degeneration (AMD), current smokers were also more likely to be visually impaired compared to never smokers (65% vs. 57%, p=0.02). |  | Smoking was linked to visual impairment (age-related disease, cataracts, AMD). Smokers were more likely to suffer visual impairment than never smokers. |

Table 6: Summary Table of Publications Relating to the Category ‘Health Signs and Symptoms’ Related to Smoking Cessation.

| First author, year | No. of subjects in the study | Country | Outcomes / concepts | Tools | Tobacco and/or nicotine product studied | Definition and stratification of smoking level | Results | Benefits of quitting | Barriers to quitting |
| --- | --- | --- | --- | --- | --- | --- | --- | --- | --- |
| Smoking cessation | | | | | | | | | |
| Borland *et al* 2010 (Borland, Yong, O'Connor, Hyland, & Thompson, 2010) | 8000 | Australia, Canada, UK,  USA | Smoking cessation, nicotine dependence | Heaviness of Smoking Index (HSI), time. | Cigarettes | Cigarettes per day were assessed by: “On average, how many cigarettes do you smoke each day, including both factory-made and roll-your-own cigarettes?” to provide a report of daily consumption. | A longer time-to-first-cigarette and fewer cigarettes smoked per day independently were associated with increasing odds of maintaining quitting attempts for at least 1 month. Combining these two factors as the Health Smoking Index did not lose predictive power. | Less dependence/ heaviness of smoking could aid maintaining cessation. | High levels of smoking could impede successful attempts at quitting. |
| Grogan *et al* 2009 (Grogan, Fry, Gough, & Conner, 2009) | 77 | UK | Aesthetics | Standard qualitative approach. | Cigarettes | Smokers vs. non-smokers. | Appearance concerns were relevant to the decision whether to start and quit smoking, as well as being linked to gender and age. | Smokers believed that smoking made them look ‘cool’, mature, and sophisticated, and would quit only if skin ageing and other negative effects on appearance became visible. | Concerns about weight gain were identified as a barrier to quitting.  Non-smokers of both genders expressed concern about yellowing of skin and teeth if they started smoking, and women non-smokers were concerned about skin ageing. |
| Bennasar Veny *et al* 2011 (Bennasar Veny et al., 2011) | 15 | Spain | Smoking cessation | Standard qualitative approach. | Cigarettes | Habitual smokers for two years | Some subjects indicated that concerns over lung cancer were real, but overall, fear of cancer was not motivation enough to give up smoking. Subjects would wait for manifestations of the disease before acting. Pregnancy had an impact on quitting or reduction in use. All participants felt that they should give up at some stage and several had tried and failed, which impacted on future perceptions of success. |  | Regarding health problems, the fear of lung cancer was a motivator to quit.  Feelings of being criminalized for smoking negatively affected self-esteem by feeling ‘frowned upon by others.’ |
| Schane *et al* 2013 (Schane, Prochaska, & Glantz, 2013) | 52 | USA | Smoking cessation, harm to self and others | Biochemical validation; 7-day point prevalence abstinence scale. | Cigarettes | At least 100 cigarettes in their lifetime, smoked at least once in the past seven days but not on every day | Results showed increases over time with motivation to quit but not in perceived difficulty of staying abstinent. Most participants reported trying to reduce or quit smoking between the counseling intervention and 3-month follow-up with no signiﬁcant difference by group. Those that were abstinent went 'cold turkey'. There was a treatment effect on quitting, not on reduction in cigarettes amongst the continuing smokers. | Social smokers felt immune from the personal health effects of tobacco but were concerned about the consequences of their second-hand smoking on others. | Non-daily smokers were a rapidly growing group, many of whom do not consider themselves smokers. |
| Bommele *et al* 2014 (Bommelé et al., 2014) | 63 | Netherlands | Smoking cessation; social environment | Standard qualitative approach  FTND | Cigarettes | Current or former hard-core smokers. Previous studies identified three basic characteristics of hard-core smokers: relative high tobacco consumption, little intention to quit, and resilience to societal pressures as indicated by a relatively long smoking history. | Pros and cons of smoking and smoking cessation were grouped into six main categories: finance, health, intrapersonal processes, social environment, physical environment, and food and weight. Although the perceived pros and cons of smoking in hard-core smokers largely mirrored the perceived pros and cons of quitting, there were some major differences with respect to weight, social integration, health of children, and stress reduction, which should be taken into account in clinical settings and when developing interventions.  Interestingly, participants tended to focus more on short-term health consequences than on long-term health effects of smoking. | Many subjects stated that ‘odors’ and ‘safety’ were key motivators to quit. Most arguments here focused on the smell smoking causes on clothing, in the home or car. ‘Safety’ arguments related to the dangers of causing fire.  Also, issues related to the social environment, particularly in the context of children and social exclusion, were considered as motivators to quit. Arguments here involved the perceived influence others have on smokers and vice versa. The subcategory ‘children’ entailed arguments about one’s own children, as well as the children of others. | Many pros of smoking (e.g., feelings of pleasure) were also mentioned as a con of quitting (e.g., missing moments of pleasure).  Many mentioned the reduction of stress as an important motivator to smoke and hence a barrier to quitting.  Benefits related to social ingratiation and smoking were seen as a barrier to quitting. |
| Bush *et al* 2014 (Bush, Hsu, Levine, Magnusson, & Miles, 2014) | 29 | USA | Weight gain, smoking cessation | Standard qualitative interview approach. | Cigarettes | Obese smokers.  Callers to a quitting service invited to take part if they smoked more than 5 cigarettes per day and over 18 years. | Participants’ awareness of weight gain associated with quitting was based on prior experience or observation of others who quit. Most viewed cessation as their primary goal and discussed other challenges as being more important than their weight, such as managing stress or coping with a chronic health condition. Although weight gain was viewed as less important than quitting, many talked about changes they had made to mitigate the anticipated weight gain. | Having a chronic disease and worries about long-term health effects of both smoking and obesity provided important external motivations for participants to change their behaviors (e.g., doctors’ advice about smoking and/or their weight). | Managing anger and irritability during the quit process was reported to be a bigger concern than weight for some subjects. |
| Carpenter *et al* 2010 (Carpenter & Gray, 2010) | 31 | USA | Smoking cessation/ motivation to quit | Weekly assessment of dependence, withdrawal and self-efficacy | Cigarettes, smokeless tobacco lozenges | Daily smokers of at least 10 cigarettes per day for at least one year. Smokers uninterested in quitting within the next month. | Use of smokeless tobacco lozenges (Ariva®/Stonewall®) led to a significant reduction in cigarettes per day, and no significant increase in total tobacco use. Significant increases in readiness to quit as well as in self-efficacy to quit smoking were also observed. | Most smokers viewed smokeless lozenges as having a more favorable safety profile compared to cigarettes. |  |
| Thompson *et al* 2016 (Thompson et al., 2016) | 99 | UK | General health, smoking cessation | FTND, expired air CO; self-reported physical activity (7-day recall); objectively assessed physical activity (Actigraph GT3X); self-reported physical activity. | Cigarettes | Disadvantaged moderate to heavy smokers who wanted to reduce but not quit smoking (without use of NRT) | A targeted intervention for economically disadvantaged individuals was compared to usual care. More participants in the intervention arm (35.5%) than in the control arm (9.7%) made a quit attempt at any point in the study (odds ratio [OR]: 5.05, 95% confidence interval (CI): 1.10; 23.15), and a greater number of participants in the intervention arm (63.3%) compared to the control arm (32.3%) achieved at least a 50% reduction in smoking at 16 weeks.  Reduction in cigarette consumption was also observed. The number of self-reported cigarettes smoked per day (−5.14 [−9.09; −1.22]) and FTND (−1.56 [−2.68; −0.43]) were decreased in the intervention group. | A smoking reduction intervention for economically disadvantaged smokers that involved personal support to increase physical activity was more effective than usual care in achieving reduction and may promote cessation |  |
| Vidrine *et al* 2009 (Vidrine et al., 2009) | 158 | USA | Smoking cessation and mental health | Wisconsin Inventory of Smoking Dependence Motives (WISDM-68); Heaviness of Smoking Index (HSI); FTND; Wisconsin Smoking Withdrawal Scale (WSWS); Self-Efﬁcacy Scale 2; Affective Information Processing Questionnaire  (AIPQ); Kentucky Inventory of Mindfulness Skills (KIMS); Mindfulness Attention Awareness Scale (MAAS) | Cigarettes | Tobacco cigarette smokers who smoked >5 cigarettes per day for the preceding year and who were motivated to quit within next 30 days. | The degree of mindfulness is signiﬁcantly associated with a combination of factors that strongly predict relapse among smokers enrolled in a cessation trial - level of nicotine dependence, withdrawal, and self-agency. | Greater degree of mindfulness was associated with lesser nicotine dependence severity. |  |
| Akturk *et al* 2012 (Akturk et al., 2012) | 50 | Turkey | Cardio-vascular disease | Standard laboratory methods | Cigarettes | Smokers (vs. non-smokers) were defined as subjects who had been smoking a minimum of ten or more cigarettes per day for at least 3 years and had never quit smoking. For the smokers, the amount of smoking was calculated by multiplying the number of cigarettes (defined by pack) smoked per day with the duration of smoking (defined by years) and expressed as pack-years. | The authors demonstrated the relationship between inter- and intra-atrial electromechanical delay and P-wave dispersion (PWD). These parameters may be useful predictive markers for the development of AF in the asymptomatic period before cardiac rhythm disturbances occur. | This finding suggests that quitting smoking may help reduce the risk of cardiovascular disease. |  |

Table 7: Summary Table of Publications Relating to the Category ‘General Health Perceptions.’

| First author, year | No. of subjects in the study | Country | Outcomes / concepts | Tools | Tobacco and/or nicotine product studied | Definition and stratification of smoking level | Results | Benefits reported | Harms reported |
| --- | --- | --- | --- | --- | --- | --- | --- | --- | --- |
| General Health Perceptions and Cigarettes | | | | | | | | | |
| Bennasar Veny *et al* 2011 (Bennasar Veny et al., 2011) | 15 | Spain | Smoking cessation | Standard qualitative approach. | Cigarettes | Habitual smokers for two years | Some subjects indicated that concerns over lung cancer were real, but overall, fear of cancer was not motivation enough to give up smoking. Subjects would wait for manifestations of the disease before acting. Pregnancy had an impact on quitting or reduction in use. All participants felt that they should give up at some stage and several had tried and failed, which impacted on future perceptions of success. |  | Regarding health problems, the fear of lung cancer was a motivator to quit.  Feelings of being criminalized for smoking negatively affected self-esteem by feeling ‘frowned upon by others.’ |
| Karadoğan *et al* 2017 (Karadoğan, Önal, Şahin, Yazıcı, & Kanbay, 2017) | 327 | Turkey | QoL, nicotine dependence | Sociodemo-graphic data form (self-report);  World Health Organization Quality of Life-bref (WHOQL-bref) questionnaire; FNTD (current smokers). | Cigarettes | Smoking status was classified as never smoker, former smoker, or current smoker. Someone who has smoked greater than 100 cigarettes in their lifetime and has smoked in the last 28 days were classified as current smoker; someone who has smoked greater than 100 cigarettes in their lifetime but has not smoked in the last 28 days were classified as former smoker; someone who has not smoked greater than 100 cigarettes in their lifetime and do not currently smoke were classified as never smoker. | Perceptions of QoL and health status scores were lower in the current smoker group compared to the never smokers (p<0.05). However, other QoL domains were not significantly different among smoking groups. Also, none of the QoL domains differed significantly according to current smokers’ nicotine dependence level. |  | Smoking cigarettes was associated with impaired health status and perceptions of QoL. |
| Grogan *et al* 2009 (Grogan et al., 2009) | 77 | UK | Aesthetics | Standard qualitative approach. | Cigarettes | Smokers vs. non-smokers. | Appearance concerns were relevant to the decision whether to start and quit smoking, as well as being linked to gender and age. | Smokers believed that smoking made them look ‘cool’, mature, and sophisticated, and would quit only if skin ageing and other negative effects on appearance became visible. | Concerns about weight gain were identified as a barrier to quitting.  Non-smokers of both genders expressed concern about yellowing of skin and teeth if they started smoking, and women non-smokers were concerned about skin ageing. |
| Mohammadnezhad *et al* 2015 (Mohammadnezhad, Tsourtos, Wilson, Ratcliffe, & Ward, 2015) | 20 | Greece, Australia | General health; lung function, attitudes | Standard qualitative approach. | Cigarettes | Smoker defined as smoking more than 100 cigarettes in their lifetime.  Smokers where a previous quit attempt had been made. | Participants had a low level of knowledge about the harmfulness of smoking as well as the benefits of smoking cessation. They exhibited a positive attitude to smoking. Influences on smoking were related to personal experience, life events and habit. Barriers to quitting included low self-confidence, family challenges, stress and loneliness. | Smoking was considered a means to overcome stress. A few cigarettes per day not considered to be harmful and therefore unlikely to lead to quitting attempts. Participants had a positive attitude to smoking as a part of social activities. | Symptoms such as phlegm, breathing problems and cough were reported but not necessarily linked directly to smoking by participants. Also, older people considered that they would not benefit from quitting (i.e. too late to change). |
| Muir *et al* 2016 (Muir & Marshall, 2016) | 80 | New Zealand | Smoking cessation/reduction; general health; nicotine; dependence | QoL SF36 | Cigarettes | Focus on smoking cessation | Four main themes emerged from the interviews with incarcerated persons: increased exercise tolerance with improvements in general health, improved palate, acknowledgment of stress and the reasoning behind beginning smoking. Lung age tests showed that most prisoners had a lung age older than their chronological age. | Many incarcerated persons considered tobacco smoking healthy when compared to alternatives (aggression, sleep deprivation, anxiety, and anger that they experience without smoking). Smoking was seen as a way to relieve the monotony and boredom of being in prison. Smoking cessation implemented prior to release of prisoners had a positive impact on health outcomes. | Smoking had a negative impact on lung age. |
| McCann *et al* 2010 (McCann, 2010) | 350,000 | USA | Personality type; quality of life; general health | Smoking prevalence was based on 2008 Behavioral Risk Factor Surveillance System (BRFSS) (2009) data; subjective well-being was the Gallup-Healthways Well-Being Index (WBI); personality variables used the Big Five framework | Cigarettes | Current smokers. | Well-being and smoking prevalence were negatively correlated and remained so when the Big Five personality states, socioeconomic status, population demographics, urban status and median age were controlled in a partial correlation. |  | Subjective well-being as assessed by WBI was impaired in smokers compared to non-smokers. |
| Schane *et al* 2013 (Schane et al., 2013) | 52 | USA | Smoking cessation, harm to self and others | Biochemical validation; 7-day point prevalence abstinence scale. | Cigarettes | At least 100 cigarettes in their lifetime, smoked at least once in the past seven days but not on every day | Results showed increases over time with motivation to quit but not in perceived difficulty of staying abstinent. Most participants reported trying to reduce or quit smoking between the counseling intervention and 3-month follow-up with no signiﬁcant difference by group. Those that were abstinent went 'cold turkey'. There was a treatment effect on quitting not on reduction in cigarettes amongst the continuing smokers. | Social smokers felt immune from the personal health effects of tobacco but were concerned about the consequences of their second-hand smoking on others. | Non-daily smokers were a rapidly growing group, many of whom do not consider themselves smokers. |
| General Health Perceptions and ‘Other’ TNPs | | | | | | | | | |
| Carpenter and Gray 2010 (Carpenter & Gray, 2010) | 31 | USA | Smoking cessation/ motivation to quit | None reported. | Cigarettes; smokeless tobacco lozenges (Ariva®/ Stonewall®) | Daily smokers of at least 10 cigarettes per day on average for at least one year. Smokers uninterested in quitting | Ariva®/Stonewall® use led to a significant reduction in cigarettes per day among subjects who switched, with no significant increases in total tobacco use. Regarding cessation, there were significant increases in two measures of readiness to quit (in the next month or within the next 6 months) as well as significant increases in self-efficacy to quit smoking. | Most smokers viewed the smokeless tobacco lozenges as safer than cigarettes (i.e. lozenges viewed as a cessation aid). |  |
| Kim *et al* 2017 (Jinyoung Kim & Lee, 2017) | 30 | South Korea | Smoking cessation/ reduction; perceptions of smoking and/or cessation; general health | FTND | E-cigarettes, waterpipe tobacco, rolling tobacco | Currently smoking, average daily cigarette consumption, total smoking period, whether they had ever tried quitting, type of NTTP tried, type of NTTP currently used, the satisfaction level of using the NTTP, and nicotine dependence. | Use of e-cigarettes was driven by increased tobacco costs, to help stop smoking and in situations where smoking was banned. Cigarette smokers were using both types of smoking devices dependent on the situation. Use of e-cigarettes did not appear to result in reduced nicotine intake. | Usage of e-cigarettes was linked to perceived improved health over cigarette use (i.e. a healthier alternative to cigarette smoking). The availability of different flavors was reported as enjoyable. ‘Second hand’ vaping was considered less harmful than smoke from cigarettes. | E-cigarettes were not reducing nicotine intake and therefore potentially not impacting on long-term health consequences. The range of enticing flavors were encouraging non-smokers to take up vaping, which may result in health consequences over time. |
| Martinasek *et al* 2013 (Martinasek, McDermott, & Bryant, 2013) | 369 | USA | Intentions and attitudes to smoking | Theoretical framework – Theory of Reasoned Action (TRA). | Hookah | Ever smokers were persons who had tried at least one puff of hookah in their lifetime; persons categorized as current smokers had smoked hookah in the previous 30 days. | Three domains emerged related to hookah smoking: benefits, negative health effects, meeting expectations. Attitudes toward hookah smoking were more positively correlated with intention to smoke than was the subjective norm. | Social benefits of hookah smoking were considered very strong, particularly for those trying to adapt to new environment, e.g., college. Hookah smoking was considered safer than smoking cigarettes. |  |
| McDonald *et al* 2015 (McDonald & Ling, 2015) | 87 | USA | General health; social integration/stigma; use of tobacco products; new devices as reduction/ cessation aids; second- hand smoke | Standard qualitative approach and semi structured interviews. | E-cigarettes | Daily, non-daily smokers, and non-smokers. | Participants used their own bodily sensations way to gauge potential risks and beneﬁts of e-cigarettes. Young adults, with their culture of personal technologies, perceived e-cigarettes as one more ‘toy’ among others, integrated into their everyday lives. | E-cigarettes were perceived as harmless, feeling ‘cleaner’ than cigarettes. Their use was in part due to attempts at quitting smoking.  Regulations and laws on smoking and perceptions around reduced nicotine addiction were also reported. |  |
| Soneji *et al* 2018 (Soneji, Sung, Primack, Pierce, & Sargent, 2018) | Population level | USA | Life years lost | Quantitative modeling approach. | E-cigarettes | Current smokers; smokers using e-cigarette as cessation tool; transition to long-term cigarette smoking among never smokers. | Modeling using available data and estimates indicated that 3.6 million adolescent non-smokers would take up use of e-cigarettes and a sizeable proportion would move onto cigarettes. E-cigarette use in 2014 represented a population level harm of about 1.6 million years of life lost over the lifetime of all adolescent and young adult never-cigarette smokers and adult current cigarette smokers. |  | E-cigarette use was considered to represent more population-level harms than benefits. |
| Rooke *et al* 2016 (Rooke, Cunningham-Burley, & Amos, 2016) | 64 | Scotland | E-cigarettes, mental health and behavior, nicotine dependence | Standard qualitative approach. | E-Cigarettes / cigarettes | Smokers and recent ex-smokers | Ambiguity around e-cigarette use in public was a concern of smokers, with differing understandings of their benefits and harms. E-cigarettes were considered a short-term tool especially in smoking cessation. | Smoking was considered to aid coping with stress. Cigarettes were reported to be enjoyable and therefore some interviewees were less likely to quit. E-cigarettes were considered safer or healthier than cigarettes. Those using e-cigarettes did so as they were concerned about their health and therefore switched away from cigarettes. | Smoking was considered to be an addiction/ habit. Health was the principal reason to stop smoking due to concerns in the long term.  Concerns were voiced around the nicotine content of e-cigarettes. Other downsides of e-cigarettes included level of ability to reduce stress, potential overuse, worry about dependence and a threat to smoking cessation. |
| Rahman *et al* 2015 (Rahman et al., 2015) | 1812 | Bangla-desh | Social integration, oral health | Qualitative semi structured interviews | Cigarettes; smokeless tobacco (SLT) | Non-smokers were defined as never smokers or ex-smokers who had not smoked a single puff in the past 10 years. | Family members’ influence was the main factor for initiation. The participants believed that people continued using SLT because of addiction (52%) and as a part of their lifestyle (23%). The majority of participants (77%) did not mention any benefit, but SLT users considered it to be a remedy for toothache (p<0.05). Almost all participants mentioned that SLT was harmful and causes heart disease, cancer, and tuberculosis. | The users believed that SLT use helped them: relieved toothache (11%), enhanced taste perceptions (6%), helped in digestion (3%), made the teeth harder (3%), and relieved anxiety (1%). | The harmful effects of SLT use, apart from the fact that it caused diseases, were: waste of money, causing harm to the environment (users spitting chewed betel leaf and/or SLT products), causing social embarrassment, family conflicts, and conflicts with religious beliefs. The majority (77%) of participants believed that there were no benefits of using SLT products. |
| Sherratt *et al* 2016 (Sherratt, Newson, Marcus, Field, & Robinson, 2015) | 20 | England | Smoking cessation, health | Standard qualitative approach. | E-cigarettes | Used to quit | Participants, particularly never users, were concerned regarding e-cigarette efﬁcacy and safety, with some evidence of misunderstanding. However, there was a perception of them being less harmful than cigarettes. Others saw e-cigarettes as a continuation of smoking and not quitting due to the hand-to-mouth action. | A number of ever users (n=3) found e-cigarettes to be beneﬁcial in reducing cravings and sustaining abstinence from tobacco smoking. E-cigarettes were also seen as an option for quitting smoking.  E-cigarette users perceived them to be safer than smoked tobacco in terms of health but never smokers were more cynical about their safety. | Some users of e-cigarettes believed their use may impede their intention to quit smoking. However, participants associated e-cigarettes with: (1) behaviors linked with tobacco smoking (e.g., mimicking aspects of the physical experience of smoking); (2) nicotine addiction; and (3) long-term use i.e. becoming reliant. |
| Wadsworth *et al* 2016 (Wadsworth, Neale, McNeill, & Hitchman, 2016) | 30 | UK | Social integration | Capability, opportunity, motivation and behavior (COM-B) theory. | E-cigarettes | Smokers and former smokers (i.e. they had not smoked within at least the last 7 days). | E-cigarette initiation (behavior) was facilitated by: capability (physical capability to use an e-cigarette and psychological capability to understand that using e-cigarettes was less harmful than smoking); opportunity (physical opportunity to access e-cigarettes in shops, at a lower cost than cigarettes, and to vape in “smoke-free” environments, as well as social opportunity to vape with friends and family); and motivation (automatic motivation including curiosity, and reﬂective motivation, including self-conscious decision-making processes related to perceived health beneﬁts). | E-cigarette adoptions was associated with a perceived reduction in harm to health, less effect on exercise compared to smoking, breaks from the ‘dirt’ of cigarettes and ability to use in public places. They were also regarded as a potential smoking cessation tool. | There was a lack of evidence on safety and effectiveness. The hand-to-mouth action was considered too similar to that of smoking cigarettes. |

Table 8: Summary Table of Publications Relating to the Category ‘Quality of Life (QoL), Health-Related Quality of Life (HRQoL), and Functional Status.’

| First author, year | No. of subjects in the study | Country | Instrument(s) used | Tobacco and/or nicotine product studied | Definition and stratification of smoking level | Results relevant to QoL/HRQoL/Functional Status | Positive effects | Negative effects |
| --- | --- | --- | --- | --- | --- | --- | --- | --- |
| Quality of Life (QoL), Health-Related Quality of Life (HRQoL), and Functional Status | | | | | | | | |
| Becona *et al* 2013 (Becoña et al., 2013) | 712 | Spain | SF-12v2 Health Survey incorporating the Physical Component Summary (PCS) and the Mental Component Summary (MCS); FTND; Stages of Change Questionnaire (motivation). | Cigarettes | Smokers vs. non-smokers. | Nicotine dependence in otherwise healthy individuals was not associated with the impairments in the physical dimension of HRQoL. However, using the MCS, nicotine-dependent smokers showed worse HRQoL than both never smokers (p=0.004) and non-nicotine dependent smokers (p=0.014). |  | Smoking impaired HRQoL related to mental well-being. |
| Karadoğan *et al* 2017 (Karadoğan et al., 2017) |  | Turkey | Sociodemographic data form (self-reported); World Health Organisation Quality of Life-bref (WHOQL-bref) questionnaire; FNTD. | Cigarettes | Current smoker: smoked over 100 cigarettes in their lifetime and smoked in the preceding 28 days; former smoker: smoked over 100 cigarettes in their lifetime but has not smoked in the preceding 28 days; never smoker: not smoked more than 100 cigarettes in their lifetime and were not currently smoking. | Factors affecting school teachers’ smoking status and quality of life were measured. Perceptions of QoL and health status scores were lower among current smokers compared to those who never smoked. |  | Smoking was associated with impaired QoL and health status. |
| Kulasekaran *et al* 2015 (Kulasekaran et al., 2015) | 186 | Germany | German-translated Tobacco Quality of Life Impact Tool (TQOLITv1). | Cigarettes; reduced toxicant prototype (RTP) | Smokers and ex-smokers. | Over a six-month period, subjects smoking either cigarettes or RTPs experienced worsening in most HRQoL measures, as well as changes in general and smoking-specific HRQoL. The impairments were less in the group using the RTP. Significant predictors for smoking-specific HRQoL impact were change in cigarette consumption, years of smoking, education and ethnicity. | Switching to the RTP from cigarettes lessened the observed impairments in HRQoL over time. |  |
| Lynch *et al* 2011 (Lynch et al., 2011) | 351 | USA | Parenting Stress Index. | Cigarettes | Non-smokers; light smokers (average of ≤14 cigarettes/day); heavy smokers (average of 15 or more cigarettes/day). | Smoking during pregnancy predicted maternal reports of parenting stress at six months postpartum; maternal psychological symptoms mediated the relationship between smoking during pregnancy and mothers’ experience of parenting stress. |  | Maternal smoking was associated with increased stress during the immediate post-partum period. |
| Martin and Sayette 2018 (Martin & Sayette, 2018) | Literature review of 13 publica-tions | Inter-national | Profile of Mood States (POMS); Self-report inventory; BSKE-30. | Cigarettes, gum, patch, nasal spray |  | When smoking, participants felt less anxious/nervous, less misunderstood, more listened to, and more successful in changing the other person’s opinion. However, smoking did not change other aspects of self-reported mood (e.g., angry, sad/depressed, interested, or pleasant) or overall emotional arousal, suggesting the significant positive social effects were not because of global affect changes. | Smoking helped reduce some aspects of negative mood states (anxiety, some aspects of social engagement) among some individuals consuming nicotine products. |  |
| Mattey *et al* 2011 (Mattey et al., 2011) | 612 | UK | Bath ankylosing spondylitis (AS) Disease Activity Index; Bath AS Functional Index (BASFI); numerical rating scale (NRS) of pain; AS quality of life questionnaire (ASQoL); evaluation of AS quality of life measures (EASi-QoL). | Cigarettes | Population based assessment; smoking criteria applied on analysis. | HRQoL was significantly worse in those patients with AS who were current or former smokers compared to those who had never smoked. |  | Smoking impaired AS-related QoL |
| McCann *et al* 2010 (McCann, 2010) | 350,000 | USA | Behavioral Risk Factor Surveillance System (BRFSS) (2009); Gallup-Healthways Well-Being Index (WBI); personality variables conceptualized within the Big Five framework. | Cigarettes | Current smokers. | Well-being was inversely correlated with smoking prevalence. |  | Well-being was impaired in subjects who smoke. |
| Sales *et al* 2009 (Sales, Oliveira, Mattos, Viana, & Pereira, 2009) | 60 | Brazil | Medical Outcomes Study 36-item Short Form Health Survey (SF-36) questionnaire; change model devised by Prochaska & DiClemente; Fagerström test for nicotine dependence (FTND) score. | Cigarettes | Smokers in a smoking cessation program. | Among participants in a smoking cessation program (bupropion and nicotine replacement), self-reported QoL scores were significantly higher among the 40 quitters than among the 20 non-quitters. | Cessation of smoking led to improvements in general HRQoL. | Smoking impaired overall HRQoL. |
| Schnoll *et al* 2013 (Schnoll, Goren, Annunziata, & Suaya, 2013) | 50,000 | USA | Fagerström Test of Nicotine Dependence (FTND); Heaviness of Smoking Index (HSI); time-to-ﬁrst-cigarette (TTFC); SF-12; SF-6D; Work Productivity and Activity Impairment (WPAI). | Cigarettes | Current smokers. | High nicotine dependence, measured by FTND, HSI or TTFC, was associated signiﬁcantly with reduced mental and physical QoL, reduced work-place productivity and more health-care use. |  | High levels of smoking were associated with impaired mental and physical QoL. |

Table 9: Summary Table of Publications Relating to the Category ‘Individual Characteristics.’

| First author, year | No. of subjects in the study | Country | Tobacco and/or nicotine product studied | Definition and stratification of smoking level | Impact of personal characteristics on use of TNPs |
| --- | --- | --- | --- | --- | --- |
| Individual Characteristics | | | | | |
| Abu-Helalah *et al* 2015 (Abu-Helalah et al., 2015) | 874 | Jordan | Hookah, cigarettes | No pre-defined smoking criteria | Data were collected using a piloted instrument based on the Global Adult Tobacco Questionnaire. The prevalence of regular cigarette smoking was 51.9 % among males and 14.1 % among females, while the prevalence of irregular cigarette smoking was 7.2 % among males and 9.2 % among females. The prevalence of heavy hookah smoking was 6.8 % for males and 6.7 % for females. Living alone was also associated with higher rates of regular smoking (39.1 %), heavy hookah smoking (20.0 %), and regular smoking with heavy hookah smoking (12.5 %) when compared to the remaining participants. Gender, age, and having a family member with cancer, were significant predictors of cigarette smoking status. |
| Aubin *et al* 2010 (Aubin et al., 2010) | 3889 | France | Cigarettes, cigar, cigarillo, pipe | Current smokers, ex-smokers, or never smokers. | Forty percent of smokers declared they intended to quit smoking permanently. Main reasons were cost (65%), physical fitness improvement (53%), fear of a future smoking-related disease (43%), weariness of tobacco (34%), and social pressure (30%). Most (62%) of those trying to quit stated that they did not use support. |
| Grogan *et al* 2009 (Grogan et al., 2009) | 87 | Singapore | Cigarettes | Smokers vs. non-smokers. | Weight gain after quitting was a significant concern for the young (i.e. 17-year-old) women in the interviewed cohort. Women non-smokers were concerned about the effects of smoking on skin ageing. |
| Kralikova *et al* 2013 (Kralikova, Novak, West, Kmetova, & Hajek, 2013) | 1738 | Poland | Cigarettes,  e-cigarettes (ECs) | Current smokers. | Comparing the participants who tried ECs but did not become regular users (n=705) and those who did become regular users (n=158), regular EC users were older (34 years vs. 31 years), were smoking currently fewer cigarettes per day (11 vs. 13), were more likely to have been pleasantly surprised by the experience of ECs (68.5% vs. 20%),and less likely to have been influenced by their early EC experience (2% vs. 46%). Overall, 18% of smokers who tried ECs became regular users. |
| McCann *et al* 2010 (McCann, 2010) | 350,000 | USA | Cigarettes | Current smokers. | Overall well-being was the prime predictor of smoking prevalence. Socioeconomic status and neuroticism were the prime predictors of well-being. Openness to experience was the sole personality or demographic variable to account for differences in smoking prevalence when well-being was controlled. |
| Memon *et al* 2016 (Memon et al., 2016) | 11 | England | Not stated | Smokers and ex-smokers. | Alcohol consumption increased the likelihood of smoking. Craving a cigarette after food and first thing in the morning were also hurdles to overcome among those attempting to quit. Additionally, among the all-female interviewees, concerns over post-cessation weight gain and the impact of the menstrual cycle were considered barriers to quitting. |
| Oh *et al* 2010 (Oh et al., 2010) | 5,000 | Europe (France, Ireland, Italy, Czech Republic, Sweden) | Cigarettes | No pre-defined smoking criteria | In this all-female cohort, women who started smoking because their friends smoked or to look ‘cool’ were most likely to have started smoking at a young age. Women who started smoking to manage stress or to feel less depressed were more likely to start smoking later in life. Levels of education and income were not associated with degree of smoking. Subjects who currently or had previously smoked were more likely to be divorced or separated than those who had never smoked. |
| Yang et al, 2011 (Yang, Shiffman, Rockett, Cui, & Cao, 2011) | 4735 | China | Cigarettes | Current smoker: someone who smoked cigarettes at time of interview; daily smoker: someone who smoked every day; occasional smoker: someone who smoked on some days. | Among daily smokers, FTND scores were negatively associated with age at smoking initiation, education, and self-efficacy for quitting smoking. FTND was also negatively associated with income among occasional smokers. Smoking was found to be more common among males than females. In most cases, subjects started smoking at age 19 years or greater. |

Table 10: Summary Table of Publications Relating to the Category ‘Environmental and Social Characteristics.’

| First author, year | Number of subjects in the study | Country | Outcomes studied | Tobacco and/or nicotine product studied | Definition and stratification of smoking level | Results |
| --- | --- | --- | --- | --- | --- | --- |
| Environmental and Social Characteristics | | | | | | |
| Brown-Johnson *et al* 2015 (Brown-Johnson et al., 2015) | 956 | USA | Smoking-related stigma in subjects with mental health diagnoses. | Cigarettes | Average of 17 cigarettes per day (SD=10) for 19 years (SD=14), and had a mean FTCD of 4.67 (SD=2.22). | Felt stigma was related to social isolation and discrimination experiences. |
| Kim *et al* 2017 (Jinyoung Kim & Lee, 2017) | 30 | South Korea | Use of non-traditional nicotine products and dependence. | E-cigarettes, waterpipe tobacco, rolling tobacco | Currently smoking, average daily cigarette consumption, total smoking period, whether they had ever tried quitting, type of NTTP tried, type of NTTP currently used, the satisfaction level of using the NTTP, and nicotine dependence. | Respondents reported starting smoking new types of tobacco products (NTTPs) as a result of increasing tobacco prices or, for adolescents, through the influence of others. E-cigarettes tended to be used in environments where cigarette smoking was banned, e.g., due to tobacco control policies. When subjects needed to focus on a task or activity for a long time (e.g., playing a game or driving), they held the e-cigarette in their mouth, consequently smoking more than when they used cigarettes alone. |
| Martin and Sayette 2018 (Martin & Sayette, 2018) | 13 studies | International | Social functioning and smoking. | Cigarettes, gum, patch, nasal spray |  | Nicotine consumption was reported to enhance social functioning in smokers, whereas withdrawal from nicotine seems to disrupt social functioning |
| Nelson *et al* 2009 (Nelson, Pederson, & Lewis, 2009) | 14,000 | USA | Reasons and patterns of smoking among soldiers. | Tobacco use unspecified | Army soldiers who use tobacco, have quit using tobacco, and have relapsed after a period of tobacco abstinence. | The army was considered a ‘tobacco-friendly environment.’ Reasons to smoke included: relaxation, relieving boredom, curbing appetite, fitting in with friends, appearing to be cool, fitting with the unit. However, many did not smoke in their own homes and around family members due to concerns over second-hand smoke. |
| Nemeth *et al* 2012 (Nemeth et al., 2012) | 23 | USA | Use of smokeless tobacco. | Smokeless tobacco (ST): chewing tobacco, dry and moist snuff | Smokeless tobacco (ST) users vs. non-users. | ST use was often associated with outdoor and manual occupations as well as leisure activities. It was also reported to be used in areas where smoking was prohibited (e.g., factories or working on farms). |
| Piña *et al* 2018 (Pina, Namba, Leyrer-Jackson, Cabrera-Brown, & Gipson, 2018) | NR | NR | Smoking and the social environment. |  | Literature review | This review raised the importance of social interaction on decision-making processes related to smoking, particularly in adolescence. The authors highlighted the role of socioeconomic status (SES) in smoking, in particular that integration into lower SES networks may increase risk of smoking. The rise in e-cigarette use due to restrictions on cigarette smoking was also emphasized. They concluded that it is important to determine the environmental factors that contribute to individual differences in vulnerability to nicotine abuse. |
| Poole-Di Salvo *et al* 2010 (Poole-Di Salvo et al., 2010) | 30,668 | USA | Behavioral and emotional patterns among children of smokers. | Cigarettes | Maternal smokers. | Children living with smokers were at increased risk for emotional or behavioral problems (e.g., relationships with family members, performance at school, behavior at home). Rates of such problems increased with increasing numbers of smokers in the household, even in the absence of maternal smoking. |
| Rahman *et al* 2015 (Rahman et al., 2015) | 1812 | Bangladesh | Use of smokeless tobacco. | Cigarettes; smokeless tobacco (SLT) | Non-smokers were defined as never smokers or ex-smokers who had not smoked a single puff in the past 10 years. | Family members’ influence was the main factor for initiation of smokeless tobacco consumption. On the other hand, a doctor’s advice was the common motivating factor to quit. |
| Romijnders *et al* 2018 (Romijnders, van Osch, de Vries, & Talhout, 2018) |  |  | Perceptions of e-cigarettes | E-cigarettes |  | E-cigarettes were considered to be safer for the environment and bystanders than cigarettes. |
| Schane *et al* 2013 (Schane et al., 2013) | 52 | USA | Perceptions related to second-hand smoke and cessation. | Cigarettes | At least 100 cigarettes in their lifetime, smoked at least once in the past seven days but not on every day | Educating non-daily smokers about the dangers of second-hand smoke for others was considered to be a more powerful cessation message than traditional smoking cessation counseling about the harm smoking does to the smoker. |
| Tan *et al* 2013 (Tan, 2013) | 66 | Singapore | Social context for smoking. | Cigarettes | Current smokers. | Some respondents (in particular women) stated that they can be judged harshly by others when smoking, and in certain cultures a heightened social surveillance was reported in this context. On the other hand, smoking was reported to enable a sense of well-being and provided camaraderie between smokers. |

Table 11: Summary Table of Publications Relating to the Category ‘Biomarkers and Biological Endpoints’

| First author, year | No. of subjects in the study | Country | Outcomes / biological endpoints studied | Tools | | Tobacco and/or nicotine product studied | Definition and stratification of smoking level | Results | Benefits reported | Harms reported |
| --- | --- | --- | --- | --- | --- | --- | --- | --- | --- | --- |
| Biomarkers and Biological Endpoints | | | | | | | | | | |
| Akturk *et al* 2012 (Akturk et al., 2012) | 50 | Turkey | **Outcome**: CV disease; **Biological endpoints:** inter- and intra-atrial electro-mechanical delay and  P-wave dispersion (PWD). | Non-invasive physiological assessment | Cigarettes | | Subjects who had been smoking a minimum of ten or more cigarettes per day for at least 3 years and had never quit smoking; the amount of smoking was calculated by multiplying the number of cigarettes (defined by pack) smoked per day with the duration of smoking (defined by years) and expressed as pack-year. | The findings indicated that smokers may have an increased risk of developing atrial rhythm disturbances. |  | Smoking cigarettes could have increased risk of cardiovascular disease in the subjects studied. |
| Bot *et al* 2014 (Bot et al., 2014) | 1026 | Netherlands | **Outcome:** Mental health; **Biological endpoint: c**otinine. | DSM-IV | Cigarettes | | Self-reported smokers (>1 cigarette per week). | Subjects with then current depression and/or anxiety who smoked reported consuming a higher number of cigarettes per day (CPD) than smokers with a remitted disorder and smokers with no lifetime disorder. |  | CPD was positively associated with cotinine levels, whereas current (at the time of study) depression and/or anxiety and high body mass index was inversely associated with cotinine. These results may explain the observed higher number of cigarettes smoked and poorer cessation rates among depressed or anxious patients in the study. |
| Brotman *et al* 2014 (Brotman et al., 2014) | 40 | USA | **Outcome:** smoking status; **Biological endpoint:** vaginal mircobiota. | Behavioral survey | Cessation program  (12 weeks); Nicoderm CQ® patches. | | Current smoker: ≥10 cigarettes/ day, no reported period of smoking abstinence >3 months in the year. | Smokers had a significant trend for increasing saliva cotinine and carbon monoxide (CO) exhalation with elevated Nugent scores, indicating greater risk of infection. Also, smokers tended to have higher Nugent Gram-stain scores and higher vaginal pH indicative of a bacterial vaginosis (BV) diagnosis compared to non-smokers. Implementing the 12-week cessation program led to a reduction in BV risk. | Smoking cessation could have helped reduce the risk of BV in some participants. | Risk of BV was significantly associated with smoking status. |
| Javed *et al* 2017 (Javed et al., 2017) | 94 | Saudi Arabia | **Outcome**: Oral health;  **Biological endpoint**: bleeding on probing. | Standardized  oral health–related questionnaire; clinical check. | Cigarettes, e-cigarettes | | Cigarette smokers (CSs); individuals exclusively vaping e-cigarettes (EC); never-smokers (NSs). | Periodontal inﬂammation and self-perceived oral symptoms were poorer among CSs than among vaping individuals and NSs. CSs exhibit signiﬁcantly greater number of sites with plaque accumulation. | Individuals who used e-cigarettes had an oral health status comparable to that of NSs. | Cigarette smoking negatively impacted oral health. |
| Lao *et al* 2009 (Lao et al., 2009) | 2999 | China | **Outcome**: Atherosclerosis;  **Biological endpoint**: inflammatory markers, C-reactive protein (CRP) and white blood cell count (WBC). | Standardized questionnaire for demographics, lifestyle, medical history, smoking history. | Cigarettes | | Never smokers, current and former smokers. Daily smoking defined as >1 per day/>7 per week; former smoker was one who used to smoke but currently not. | Smoking is associated with increased CRP and WBC levels. Smoking cessation is associated with the reduction of the increase, confirming the benefits of quitting. | CRP levels started to decline in those who had quit smoking for 5-9 years and became lower with longer cessation. With the CRP level in those who had quit>20 years being similar to never smokers. | Inflammation may be a potential mechanism by which smoking promotes atherosclerotic disease. |
| Miyatake *et al* 2010 (Miyatake, Moriyasu, et al., 2010) | 286 | Japan | **Outcome:** renal function; **Biological endpoint:** glomerular ﬁltration rate (eGFR). | Longitudinal analysis: clinical check-up and smoking evaluation. | Cigarettes | | Smokers: Number of cigarettes per day. | This study did not show any evidence for a link between eGFR and cigarette smoking. |  | Renal function (eGFR) was weakly and negatively correlated with the number of cigarettes smoked per day by cross-sectional analysis. However, longitudinal analysis showed that changes in eGFR in subjects with cigarette smoking were signiﬁcantly smaller than those in subjects without cigarette smoking. |
| Morris *et al* 2016 (Morris, Mielock, & Rao, 2016) | 64 | USA | **Outcome:** stress and dependence; **Biological endpoint:** salivary cotinine levels, exhaled carbon monoxide (CO). | DSM-IV; WHO-composite international diagnostic interview; FTQ; Hughes-Hatsukami withdrawal symptoms questionnaire; TSST. | Cigarettes | | Smoked >10 cigarettes per day in the past six months. | Recent nicotine use and lower dependence severity was associated with increased activation of the stress response systems.  In contrast, more severe levels of dependence were associated with a downregulation of stress response systems. |  | Smokers with more severe levels of nicotine dependence showed diminished cortisol responses and may have benefitted from pharmacological interventions that upregulated the hypothalamic–pituitary–adrenal HPA axis. In contrast, smokers with lower dependence severity showed greater cortisol responses and may have benefited from pharmacological interventions that downregulated HPA reactivity. |
| Nunes *et al* 2012 (Nunes et al., 2012) | 155 | Brazil | **Outcome:** mental health/ depressive state; **Biological endpoint:** inflammatory markers:  C-reactive protein (hs-CRP), tumor necrosis factor-alpha (TNF-α), and interleukin-6 (IL-6). | ICD 10 classification of depressive disorder; F33; FTND. | Cigarettes | | Smokers recruited from a smoking cessation program. | Individuals who smoked and were depressed had higher levels of inflammatory biomarkers, particularly CRP levels, TNF-α and IL-6 compared to smokers who were not diagnosed with depression. |  | Depressed smokers had worse physical health outcomes (inflammation, CV disease, lung disease) and greater work-related disability than non-depressed smokers. |
| Rollini *et al* 2014 (Rollini et al., 2014) | 60 | USA | **Outcome**:  Athero-sclerosis  **Biological endpoint:** serum cotinine levels. | Smoking questionnaire; clinical history; use of antiplatelet treatment; blood samples. | Cigarettes | | Non-smokers, light smokers, and heavy smokers according to cotinine serum levels; active smokers: >10 cigarettes/day. | In heavy smokers with vascular disease manifestations, clopidogrel use was associated with enhanced platelet inhibitory effects, affecting purinergic and non-purinergic pathways, compared to the effects of aspirin.  Moreover, among smokers, clopidogrel resulted in a trend towards enhanced effects on parameters of clot generation. |  | Smoking was a strong, independent risk factor for cardiovascular disease in this study. The choice of anti-platelet therapy appeared to modulate the level of risk of clotting. |
| Torigian *et al* 2017 (Torigian et al., 2017) | 20 males | USA | **Biological endpoint:** 18F-fluorodeoxyglucose (FDG) measuring using positron emission tomography/computed tomography (PET/CT). | FTND; mini-mental state examination (MMSE). | Cigarettes | | Chronic heavy smoking: ≥15 pack-year smoking history and no recent cessation attempts. | FDG-PET/CT revealed significantly decreased metabolic activity in the lumbar spinal bone marrow and liver, as well as increased metabolic activity of visceral adipose tissue (VAT) in chronic smokers compared to non-smokers. |  | In this study, chronic heavy smoking was associated with a lowering of metabolic activity in the bone marrow and liver. However, it was associated with increased metabolic activity in body fat tissue. |
| Ulvik *et al* 2010 (Ulvik et al., 2010) | Population study | Norway | **Outcome:** ischemic heart disease; **Biological endpoint:** selected B vitamins, cotinine. | Clinical trial retrospective analysis. | Cigarettes | | Smoking status was assessed by asking if they were current or former smokers, and for former smokers, how long ago they had quit smoking. | The highest circulating concentrations of folate and pyridoxal 5 phosphate (PLP) (all p<0.005), and lowest concentrations of total plasma homocysteine were observed for self-reported never smokers, followed by self-reported ex-smokers and current smokers | Smoking cessation had positive impact on markers of ischemic heart disease. This trend continued to increase with increasing time since smoking cessation. | Compared to never smokers, current smokers had significantly lower circulating concentrations of folate, PLP, and riboflavin, which can help reduce risk of ischemic heart disease. |
| Xie *et al* 2014 (Xie et al., 2014) | 100 | Netherlands | **Outcome**: respiratory disease/ symptoms; **Biological endpoint:**  bronchial airway wall thickness. | Clinical trial retrospective analysis. | Cigarettes | | Heavy smokers (≥ 15 cigarettes/ day during ≥ 25 years or ≥ 10 cigarettes/day during ≥ 30 years). | The group with respiratory symptoms had more current smokers than the group without symptoms. Heavy smokers with chronic respiratory symptoms had significantly thicker bronchial airway walls, which represented airway remodeling via an inflammatory process. |  | The group with respiratory symptoms had significantly worse pulmonary function and generally thicker airway walls up to the trachea than the group without symptoms. Measuring bronchial wall thickness could be useful for diagnosing early bronchitis. |

Abu-Helalah, M. A., Alshraideh, H. A., Al-Serhan, A. A., Nesheiwat, A. I., Da'na, M., & Al-Nawafleh, A. (2015). Epidemiology, attitudes and perceptions toward cigarettes and hookah smoking amongst adults in Jordan. *Environ Health Prev Med, 20*(6), 422-433. doi:10.1007/s12199-015-0483-1

Akturk, E., Yağmur, J., Açıkgöz, N., Ermi, N., Cansel, M., Karaku, Y., . . . Pekdemir, H. (2012). Assessment of atrial conduction time by tissue Doppler echocardiography and P-wave dispersion in smokers. *J Interv Card Electrophysiol, 34*(2), 247-253. doi:10.1007/s10840-011-9658-x

Aubin, H. J., Peiffer, G., Stoebner-Delbarre, A., Vicaut, E., Jeanpetit, Y., Solesse, A., . . . Thomas, D. (2010). The French Observational Cohort of Usual Smokers (FOCUS) cohort: French smokers perceptions and attitudes towards smoking cessation. *BMC Public Health, 10*(100). doi:10.1186/1471-2458-10-100

Becoña, E., Vázquez, M. I., Míguez, M. C., Fernández del Río, E., López-Durán, A., Martínez, Ú., & Piñeiro, B. (2013). Smoking habit profile and health-related quality of life. *Psicothema, 25*(4), 421-426. doi:10.7334/psicothema2013.73

Bennasar Veny, M., Pericas Beltrán, J., González Torrente, S., Segui González, P., Aguiló Pons, A., & Tauler Riera, P. (2011). Self-perceived factors associated with smoking cessation among primary health care nurses: a qualitative study. *Rev. Latino-Am. Enfermagem 19*(6), 1437-1444.

Bommelé, J., Schoenmakers, T. M., Kleinjan, M., van Straaten, B., Wits, E., Snelleman, M., & van de Mheen, D. (2014). Perceived pros and cons of smoking and quitting in hard-core smokers: a focus group study. *BMC Public Health, 14*, 175-185.

Borland, R., Yong, H. H., O'Connor, R. J., Hyland, A., & Thompson, M. E. (2010). The reliability and predictive validity of the Heaviness of Smoking Index and its two components: findings from the International Tobacco Control Four Country study. *Nicotine Tob Res., 12 Suppl 1*, S45-50. doi:10.1093/ntr/ntq038

Bot, M., Vink, J., Milaneschi, Y., Smit, J. H., Kluft, C., Neuteboom, J., & Penninx, B. (2014). Plasma cotinine levels in cigarette smokers: impact of mental health and other correlates. *Eur Addict Res, 20*(4), 183-191. doi:10.1159/000356809

Brody, A. L., Olmstead, R. E., Abrams, A. L., Costello, M. R., Khan, A., Kozman, D., . . . Mandelkern, M. A. (2009). Effect of a history of major depressive disorder on smoking-induced dopamine release. *Biol Psychiatry, 66*(9), 898-901. doi:10.1016/j.biopsych.2009.06.011

Brook, D. W., Rubenstone, E., Zhang, C., & Brook, J. S. (2012). Trajectories of cigarette smoking in adulthood predict insomnia among women in late mid-life. *Sleep Medicine, 13*(9), 1130-1137.

Brotman, R. M., He, X., Gajer, P., Fadrosh, D., Sharma, E., Mongodin, E. F., . . . Rath, J. M. (2014). Association between cigarette smoking and the vaginal microbiota: a pilot study. *BMC Infect Dis, 14*, 471-482.

Brown-Johnson, C. G., Cataldo, J. K., Orozco, N., Lisha, N. E., Hickman, N. J., 3rd, & Prochaska, J. J. (2015). Validity and reliability of the Internalized Stigma of Smoking Inventory: An exploration of shame, isolation, and discrimination in smokers with mental health diagnoses. *Am J Addict, 24*(5), 410-418. doi:10.1111/ajad.12215

Bush, T., Hsu, C., Levine, M. D., Magnusson, B., & Miles, L. (2014). Weight gain and smoking: perceptions and experiences of obese quitline participants. *BMC Public Health, 14*, 1229. doi:10.1186/1471-2458-14-1229

Caldirola, D., Cavedini, P., Riva, A., Di Chiaro, N. V., & Perna, G. (2016). Cigarette smoking has no pro-cognitive effect in subjects with obsessive-compulsive disorder: A preliminary study. *Psychiatria Danubina, 28*(1), 86-90.

Caldirola, D., Daccò, S., Grassi, M., Citterio, A., Menotti, R., Cavedini, P., . . . Perna, G. (2013). Effects of cigarette smoking on neuropsychological performance in mood disorders: a comparison between smoking and nonsmoking inpatients. *The Journal of Clinical Psychiatry 74*(2), e130-136.

Carpenter, M. J., & Gray, K. M. (2010). A pilot randomized study of smokeless tobacco use among smokers not interested in quitting: changes in smoking behavior and readiness to quit. *Nicotine & Tobacco Research 12*(2), 136-143.

Croucher, R., Haque, M. F., & Kassim, S. (2013). Oral pain before and after smokeless tobacco cessation in U.K.-resident Bangladeshi women: cross-sectional analyses. *Nicotine & Tobacco Research 15*(5), 896-903.

Depp, C. A., Bowie, C. R., Mausbach, B. T., Wolyniec, P., Thornquist, M. H., Luke, J. R., . . . Harvey, P. D. (2015). Current smoking is associated with worse cognitive and adaptive functioning in serious mental illness. *Acta Psychiatr Scand, 131*(5), 333-341.

Ditre, J. W., Zale, E. L., Heckman, B. W., & Hendricks, P. S. (2017). A measure of perceived pain and tobacco smoking interrelations: pilot validation of the pain and smoking inventory. *Cogn Behav Ther, 46*(6), 339-351.

Doiron, M., Dupré, N., Langlois, M., Provencher, P., & Simard, M. (2017). Smoking history is associated to cognitive impairment in Parkinson's disease. *Aging Ment Health, 21*(3), 322-326.

Gonzalez, A., Zvolensky, M. J., Vujanovic, A. A., Leyro, T. M., & Marshall, E. C. (2008). An evaluation of anxiety sensitivity, emotional dysregulation, and negative affectivity among daily cigarette smokers: relation to smoking motives and barriers to quitting. *J Psychiatr Res., 43*(2), 138-147.

Grogan, S., Fry, G., Gough, B., & Conner, M. (2009). Smoking to stay thin or giving up to save face? Young men and women talk about appearance concerns and smoking. *Br J Health Psychol, 14*(1), 175-186.

Hawari, F. I., Obeidat, N. A., Ghonimat, I. M., Ayub, H. S., & Dawahreh, S. S. (2017). The effect of habitual waterpipe tobacco smoking on pulmonary function and exercise capacity in young healthy males: A pilot study. *Respiratory Medicine, 122*, 71-75.

Heffernan, T. M., O'Neill, T. S., & Moss, M. (2012). Smoking-related prospective memory deficits in a real-world task. *Drug Alcohol Depend, 120*(1-3), 1-6.

Highland, K. B., & McChargue, D. E. (2011). Stress-induced cardiovascular reactivity among African American smokers. *American Journal of Health Behavior, 35*(1), 51-59.

Holley, A. L., Law, E. F., Tham, S. W., Myaing, M., Noonan, C., Strachan, E., & Palermo, T. M. (2013). Current smoking as a predictor of chronic musculoskeletal pain in young adult twins. *J Pain, 14*(10), 1131-1139.

Ichikawa, Y., Kitagawa, K., Kato, S., Dohi, K., Hirano, T., Ito, M., & Sakuma, H. (2014). Altered coronary endothelial function in young smokers detected by magnetic resonance assessment of myocardial blood flow during the cold pressor test. *The International Journal of cardiovascular Imaging, 30*, 73-80.

Javed, F., Abduljabbar, T., Vohra, F., Malmstrom, H., Rahman, I., & Romanos, G. E. (2017). Comparison of Periodontal Parameters and Self-Perceived Oral Symptoms Among Cigarette Smokers, Individuals Vaping Electronic Cigarettes, and Never-Smokers. *J Periodontol, 88*(10), 1059-1065. doi:10.1902/jop.2017.170197

Johnson, A. L., & McLeish, A. C. (2017). Differences in panic psychopathology between smokers with and without asthma. *Psychology Health & Medicine, 22*(1), 110-120.

Kahler, C. W., Daughters, S. B., Leventhal, A. M., Rogers, M. L., Clark, M. A., Colby, S. M., . . . Buka, S. L. (2009). Personality, psychiatric disorders, and smoking in middle-aged adults. *Nicotine & Tobacco Research, 11*(7), 833-841.

Karadoğan, D., Önal, Ö., Şahin, D., Yazıcı, S., & Kanbay, Y. (2017). Evaluation of school teachers’ sociodemographic characteristics and quality of life according to their cigarette smoking status: a cross-sectional study from eastern Black Sea region of Turkey. *Tuberkuloz ve Toraks, 65*(1), 18-24. doi:10.1183/1393003.congress-2017.PA2667

Kim, J., Gall, S. L., Dewey, H. M., Macdonell, R. A., Sturm, J. W., & Thrift, A. G. (2012). Baseline smoking status and the long-term risk of death or nonfatal vascular event in people with stroke: a 10-year survival analysis. *Stroke, 43*(12), 3173-3178.

Kim, J., & Lee, S. (2017). Using Focus Group Interviews to Analyze the Behavior of Users of New Types of Tobacco Products. *Journal of preventive medicine and public health, 50*(5), 336-346. doi:10.3961/jpmph.17.052

Kralikova, E., Novak, J., West, O., Kmetova, A., & Hajek, P. (2013). Do e-cigarettes have the potential to compete with conventional cigarettes?: a survey of conventional cigarette smokers' experiences with e-cigarettes. *Chest, 144*(5), 1609-1614.

Kulasekaran, A., Proctor, C., Papadopoulou, E., Shepperd, C. J., Guyer, R., Gandek, B., & Ware, J. E. (2015). Preliminary Evaluation of a New German Translated Tobacco Quality of Life Impact Tool to Discriminate Between Healthy Current and Former Smokers and to Explore the Effect of Switching Smokers to a Reduced Toxicant Prototype Cigarette. *Nicotine & Tobacco Research, 17*(12), 1456-1464. doi:<https://doi.org/10.1093/ntr/ntv024>

Lao, X. Q., Jiang, C. Q., Zhang, W. S., Adab, P., Lam, T. H., Cheng, K. K., & Thomas, G. N. (2009). Smoking, smoking cessation and inflammatory markers in older Chinese men: The Guangzhou Biobank Cohort Study. *Atherosclerosis, 203*(1), 304-310.

Lappan, S., Thorne, C. B., Long, D., & Hendricks, P. S. (2018). Longitudinal and Reciprocal Relationships Between Psychological Well-Being and Smoking. *Nicotine Tob Res*. doi:10.1093/ntr/nty185

Lynch, M. E., Johnson, K. C., Kable, J. A., Carroll, J., & Coles, C. D. (2011). Smoking in pregnancy and parenting stress: maternal psychological symptoms and socioeconomic status as potential mediating variables. *Nicotine Tob Res., 13*(7), 532-539.

Lyvers, M., Carlopio, C., Honours, V. B., & Edwards, M. S. (2014). Mood, mood regulation, and frontal systems functioning in current smokers, long-term abstinent ex-smokers, and never-smokers. *J Psychoactive Drugs, 46*(2), 133-139.

Martin, L. M., & Sayette, M. A. (2018). A review of the effects of nicotine on social functioning. *Exp Clin Psychopharmacol, 26*(5), 425-439. doi:10.1037/pha0000208

Martinasek, M. P., McDermott, R. J., & Bryant, C. A. (2013). Antecedents of university students' hookah smoking intention. *Am J Health Behav, 37*(5), 599-609.

Mattey, D. L., Dawson, S. R., Healey, E. L., & Packham, J. C. (2011). Relationship between smoking and patient-reported measures of disease outcome in ankylosing spondylitis. *J Rheumatol, 38*(12), 2608-2615.

McCann, S. J. (2010). Subjective well-being, personality, demographic variables, and American state differences in smoking prevalence. *Nicotine Tob Res, 12*(9), 895-904. doi:10.1093/ntr/ntq113

McDonald, E. A., & Ling, P. M. (2015). One of several 'toys' for smoking: young adult experiences with electronic cigarettes in New York City. *Tob Control, 24*(6), 588-593.

McLeish, A. C., Zvolensky, M. J., Del Ben, K. S., & Burke, R. S. (2009). Anxiety sensitivity as a moderator of the association between smoking rate and panic-relevant symptoms among a community sample of middle-aged adult daily smokers. *American Journal on Addictions, 18*(1), 93-99.

Melis, M., Lobo, S. L., Ceneviz, C., Ruparelia, U. N., Zawawi, K. H., Chandwani, B. P., & Mehta, N. R. (2010). Effect of cigarette smoking on pain intensity of TMD patients: a pilot study. *Cranio, 28*(3), 187-192.

Memon, A., Barber, J., Rumsby, E., Parker, S., Mohebati, L., de Visser, R. O., . . . Sundin, J. (2016). What factors are important in smoking cessation and relapse in women from deprived communities? A qualitative study in Southeast England. *Public Health, 134*, 39-45.

Mendiondo, M. S., Alexander, L. A., & Crawford, T. (2010). Health profile differences for menthol and non-menthol smokers: findings from the National Health Interview Survey. *Addiction, 105, Suppl 1*, 124-140.

Miyatake, N., Moriyasu, H., Sakano, N., Tada, S., Suzue, T., & Hirao, T. (2010). Influence of cigarette smoking on estimated glomerular filtration rate (eGFR) in Japanese male workers. *Acta Medica Okayama 64*, 385-390.

Miyatake, N., Numata, T., Nishii, K., Sakano, N., Suzue, T., Hirao, T., . . . Tabata, I. (2010). Influence of cigarette smoking on estimated glomerular filtration rate (eGFR) in Japanese male workers. *Acta Medica Okayama, 64*, 385-390.

Mohammadi, S., Mazhari, M. M., Mehrparvar, A. H., & Attarchi, M. S. (2009). Cigarette smoking and occupational noise-induced hearing loss. *The European Journal of Public Health, 20*(4), 452-455.

Mohammadnezhad, M., Tsourtos, G., Wilson, C., Ratcliffe, J., & Ward, P. (2015). "I have never experienced any problem with my health. So far, it hasn't been harmful": older Greek-Australian smokers' views on smoking: a qualitative study. *BMC Public Health, 15*, 304-315.

Morris, M. C., Mielock, A. S., & Rao, U. (2016). Salivary stress biomarkers of recent nicotine use and dependence. *Am J Drug Alcohol Abuse, 42*(6), 640-648.

Muir, S., & Marshall, B. (2016). Changes in Health Perceptions of Male Prisoners Following a Smoking Cessation Program. *J Correct Health Care, 22*(3), 247-256.

Nelson, J. P., Pederson, L. L., & Lewis, J. (2009). Tobacco use in the Army: illuminating patterns, practices, and options for treatment. *Military Medicine, 174*(2), 162-169.

Nemeth, J. M., Liu, S. T., Klein, E. G., Ferketich, A. K., Kwan, M. P., & Wewers, M. E. (2012). Factors influencing smokeless tobacco use in rural Ohio Appalachia. *J Community health, 37*(6), 1208-1217.

Nikcevic, A. V., & Spada, M. M. (2010). Metacognitions about smoking: a preliminary investigation. *Clin Psychol Psychother, 17*(6), 536-542.

Nunes, S. O., Vargas, H. O., Brum, J., Prado, E., Vargas, M. M., de Castro, M. R., . . . Berk, M. (2012). A comparison of inflammatory markers in depressed and nondepressed smokers. *Nordic Journal of Psychiatry, 14*, 540-546.

Oh, D. L., Heck, J. E., Dresler, C., Allwright, S., Haglund, M., Del Mazo, S. S., . . . Hashibe, M. (2010). Determinants of smoking initiation among women in five European countries: a cross-sectional survey. *BMC Public Health, 10*(74).

Pasco, J. A., Williams, L. J., Jacka, F. N., Ng, F., Henry, M. J., Nicholson, G. C., . . . Berk, M. (2008). Tobacco smoking as a risk factor for major depressive disorder: population-based study. *Br J Psychiatry, 193*(4), 322-326.

Pina, J. A., Namba, M. D., Leyrer-Jackson, J. M., Cabrera-Brown, G., & Gipson, C. D. (2018). Social Influences on Nicotine-Related Behaviors. *Int Rev Neurobiol, 140*, 1-32. doi:10.1016/bs.irn.2018.07.001

Poole-Di Salvo, E., Liu, Y. H., Brenner, S., & Weitzman, M. (2010). Adult household smoking is associated with increased child emotional and behavioral problems. *Journal of developmental and behavioral pediatrics, 31*(2), 107-115.

Rahman, M. A., Mahmood, M. A., Spurrier, N., Rahman, M., Choudhury, S. R., & Leeder, S. (2015). Why do Bangladeshi people use smokeless tobacco products? *Asia-Pacific Journal of Public Health, 27*(2). doi:doi: 10.1177/1010539512446957

Rollini, F., Franchi, F., Cho, J. R., Degroat, C., Bhatti, M., Ferrante, E., . . . Angiolillo, D. J. (2014). Cigarette smoking and antiplatelet effects of aspirin monotherapy versus clopidogrel monotherapy in patients with atherosclerotic disease: results of a prospective pharmacodynamic study. *Journal of Cardiovascular Translational Research 7*(1), 53-63.

Romijnders, K., van Osch, L., de Vries, H., & Talhout, R. (2018). Perceptions and Reasons Regarding E-Cigarette Use among Users and Non-Users: A Narrative Literature Review. *Int J Environ Res Public Health, 15*(6). doi:10.3390/ijerph15061190

Rooke, C., Cunningham-Burley, S., & Amos, A. (2016). Smokers' and ex-smokers' understanding of electronic cigarettes: a qualitative study. *Tob Control, 25*, e60-e66.

Roth, B., Bengtsson, M., & Ohlsson, B. (2013). Diarrhoea is not the only symptom that needs to be treated in patients with microscopic colitis. *European Journal of Internal Medicine, 24*(6), 573-578.

Sales, M. P., Oliveira, M. I., Mattos, I. M., Viana, C. M., & Pereira, E. D. (2009). The impact of smoking cessation on patient quality of life. *Jornal Brasileiro de Pneumologia, 35*(5), 436-441.

Schane, R. E., Prochaska, J. J., & Glantz, S. A. (2013). Counseling nondaily smokers about secondhand smoke as a cessation message: a pilot randomized trial. *Nicotine Tob Res., 15*(2), 334-342.

Schnoll, R. A., Goren, A., Annunziata, K., & Suaya, J. A. (2013). The prevalence, predictors and associated health outcomes of high nicotine dependence using three measures among US smokers. *Addiction, 108*(11), 1989-2000.

Segarra, R., Zabala, A., Eguíluz, J. I., Ojeda, N., Elizagarate, E., Sánchez, P., . . . Gutiérrez, M. (2011). Cognitive performance and smoking in first-episode psychosis: the self-medication hypothesis. *Eur Arch Psychiatry Clin Neurosci, 261*(4), 241-250.

Sherratt, F. C., Newson, L., Marcus, M. W., Field, J. K., & Robinson, J. (2015). Perceptions towards electronic cigarettes for smoking cessation among Stop Smoking Service users. *Br J Health Psychol, 21*(2), 421-433.

Soneji, S. S., Sung, H. Y., Primack, B. A., Pierce, J. P., & Sargent, J. D. (2018). Quantifying population-level health benefits and harms of e-cigarette use in the United States. *PLoS One, 13*(3), e0193328. doi:10.1371/journal.pone.0193328

Talati, A., Wickramaratne, P. J., Keyes, K. M., Hasin, D. S., Levin, F. R., & Weissman, M. M. (2013). Smoking and psychopathology increasingly associated in recent birth cohorts. *Drug Alcohol Depend, 133*(2), 724-732.

Tan, Q. H. (2013). Smoking spaces as enabling spaces of wellbeing. *Health Place, 24*, 173-182.

Tatullo, M., Gentile, S., Paduano, F., Santacroce, L., & Marrelli, M. (2016). Crosstalk between oral and general health status in e-smokers. *Medicine 95*(49).

Taylor, G., McNeill, A., Girling, A., Farley, A., Lindson-Hawley, N., & Aveyard, P. (2014). Change in mental health after smoking cessation: systematic review and meta-analysis. *BMJ 348*, g1151-g1151. doi:10.1136/bmj.g1151

Thompson, T. P., Greaves, C. J., Ayres, R., Aveyard, P., Warren, F. C., Byng, R., . . . Taylor, A. (2016). An Exploratory Analysis of the Smoking and Physical Activity Outcomes From a Pilot Randomized Controlled Trial of an Exercise Assisted Reduction to Stop Smoking Intervention in Disadvantaged Groups. *Nicotine & Tobacco Research, 18*(3), 289-297.

Torigian, D. A., Green-McKenzie, J., Liu, X., Shofer, F. S., Werner, T., Smith, C. E., . . . Alavi, A. (2017). A Study of the Feasibility of FDG-PET/CT to Systematically Detect and Quantify Differential Metabolic Effects of Chronic Tobacco Use in Organs of the Whole Body-A Prospective Pilot Study. *Academic Radiology, 24*, 930-940.

Ulvik, A., Ebbing, M., Hustad, S., Midttun, Ø., Nygård, O., Vollset, S. E., . . . Ueland, P. M. (2010). Long- and short-term effects of tobacco smoking on circulating concentrations of B vitamins. *Clinical Chemistry, 56*(5), 755-763.

Vidrine, J. I., Businelle, M. S., Cinciripini, P., Li, Y., Marcus, M. T., Waters, A. J., . . . Wetter, D. W. (2009). Associations of mindfulness with nicotine dependence, withdrawal, and agency. *Subst Abus, 30*(4), 318-327.

Volkman, J. E., DeRycke, E. C., Driscoll, M. A., Becker, W. C., Brandt, C. A., Mattocks, K. M., . . . Bastian, L. A. (2015). Smoking Status and Pain Intensity Among OEF/OIF/OND Veterans. *Pain Med, 16*(9), 1690-1696.

Vujanovic, A. A., Marshall-Berenz, E. C., Beckham, J. C., Bernstein, A., & Zvolensky, M. J. (2010). Posttraumatic stress symptoms and cigarette deprivation in the prediction of anxious responding among trauma-exposed smokers: a laboratory test. *Nicotine Tob Res., 12*(11), 1080-1088.

Wadsworth, E., Neale, J., McNeill, A., & Hitchman, S. C. (2016). How and Why Do Smokers Start Using E-Cigarettes? Qualitative Study of Vapers in London, UK. *Int J Environ Res Public Health, 13*(7), 661-674.

Xie, X., Dijkstra, A. E., Vonk, J. M., Oudkerk, M., Vliegenthart, R., & Groen, H. J. (2014). Chronic respiratory symptoms associated with airway wall thickening measured by thin-slice low-dose CT. *American Journal of Roentgenology, 203*(4), W383-W390.

Yang, T., Shiffman, S., Rockett, I. R., Cui, X., & Cao, R. (2011). Nicotine dependence among Chinese city dwellers: a population-based cross-sectional study. *Nicotine & Tobacco Research, 13*(7), 556-564.

Yu, A., Cai, X., Zhang, Z., Shi, H., Liu, D., Zhang, P., & Fu, Z. (2015). Effect of nicotine dependence on opioid requirements of patients after thoracic surgery. *Acta Anaesthesiologica Scandinavica, 59*(1), 115-122.

Zhang, X., Kahende, J., Fan, A. Z., Barker, L., Thompson, T. J., Mokdad, A. H., . . . Saaddine, J. B. (2011). Smoking and visual impairment among older adults with age-related eye diseases. *Prev Chronic Dis., 8*(4), A84.

Zhao, L., Xu, L., Lai, Y., Che, C., & Zhou, Y. (2012). Temporal changes of smoking status and motivation in Chinese patients with hepatitis B: relationship with anxiety and depression. *Journal of Clinical Nursing, 21*(15-16), 2193-2201.

Zorlu, N., Cropley, V. L., Zorlu, P. K., Delibas, D. H., Adibelli, Z. H., Baskin, E. P., . . . Pantelis, C. (2017). Effects of cigarette smoking on cortical thickness in major depressive disorder. *J Psychiatr Res., 84*, 1-8.
